# The C3–C3aR axis modulates trained immunity in alveolar macrophages

**DOI:** 10.7554/eLife.104977

**Published:** 2026-09-10

**Authors:** Alexander P Earhart, Alberto E Lopez, Josue I Hernandez, Aasritha Nallapu, Deebly Chavez, Sayahi Suthakaran, Brian Yang, Jungheun Hyun, Rafael Aponte Alburquerque, Marick Starick, Lorena Garnica, Ayse Naz Ozanturk, Rahul Kumar Maurya, Xiaobo Wu, Jeffrey Haspel, Jae Woo Lee, Jaime Hook, Hrishikesh S Kulkarni

**Affiliations:** 1 John T. Milliken Department of Medicine, Washington University School of Medicine St. Louis United States; 2 Department of Medicine, University of California Los Angeles David Geffen School of Medicine Los Angeles United States; 3 Lung Imaging Laboratory, Division of Pulmonary, Critical Care, & Sleep Medicine, Department of Medicine, Icahn School of Medicine at Mount Sinai New York United States; 4 Department of Anesthesia, University of California, Los Angeles David Geffen School of Medicine Los Angeles United States; 5 Department of Microbiology, Icahn School of Medicine at Mount Sinai New York United States; 6 Department of Stem Cell Biology & Regenerative Medicine, Icahn School of Medicine at Mount Sinai New York United States; 7 Global Health and Emerging Pathogens Institute, Icahn School of Medicine at Mount Sinai New York United States; Murdoch Childrens Research Institute Australia; National Institute of Immunology India

**Keywords:** complement, alveolar macrophages, training, *Pseudomonas*, *Candida*, immunometabolism, Human, Mouse

## Abstract

Complement protein C3 is crucial for immune responses in mucosal sites such as the lung, where it aids in microbe elimination, and enhances inflammation. While trained immunity – enhanced secondary responses of innate immune cells after prior exposure – is well-studied, the role of the complement system in trained immune responses remains unclear. We investigated the role of C3 in trained immunity and found that alveolar macrophage (AM) *C3* and *C3aR1* expression increased in humans after an intranasal exposure to a training stimulus. In vivo, trained wild-type mice showed significantly elevated proinflammatory cytokines and increased C3a levels upon a second stimulus. Ex vivo, trained C3-deficient AMs displayed reduced chemokine and cytokine output as well as impaired phagocytosis and reactive oxygen species production compared to wild-type AMs. Real-time confocal microscopy of live, intact mouse alveoli revealed that AMs internalize C3 rapidly after alveolar microinstillation, as compared to C3a. Correspondingly, the blunted cytokine output was restored by exogenous C3 but not by C3a. Inhibiting C3aR, both pharmacologically and with a genetic C3aR knockout, prevented this restoration, indicating the necessity of C3aR engagement. Mechanistically, trained WT AMs demonstrated enhanced glycolytic activity compared to C3-deficient AMs – a defect corrected by exogenous C3 in a C3aR-dependent manner. These findings reveal that C3 modulates trained immunity in AMs through C3aR signaling and highlight a novel role for C3 in trained immunity.

## Introduction

The complement system is a crucial part of immunity, consisting of proteolytic enzymes that generate fragments which enhance antibody binding and help induce phagocytosis (Sahu et al., 2022). A central component of this system is the C3 protein, which is cleaved into its constituent components C3a and C3b upon activation (Kulkarni et al., 2018). C3b tags pathogens for phagocytosis and plays a vital role in the formation of the C3 convertase, which cleaves C5 and eventually forms the membrane attack complex that lyses targeted cells (Janssen et al., 2006). Meanwhile, C3a acts as an anaphylatoxin, binding to its cognate C3a receptor (C3aR) on and within cells, and modulates inflammatory responses (Kildsgaard et al., 2000). C3 activation has also been shown to favor an increase in glycolysis, suggesting a plausible mechanism for enhanced inflammatory activity (Friščić et al., 2021; Kang et al., 2024b). Moreover, C3 is present at low levels in the bronchoalveolar lavage (BAL) fluid of uninjured mice as well as humans, indicative of localized production (Bolger et al., 2007). This local C3 production increases during injury, affecting mucosal responses to infection (Bolger et al., 2007). Additionally, the conversion of C3 to a conformationally altered C3(H_2_O) moiety increases during inflammation (Elvington et al., 2019). This C3(H_2_O) form is that which is internalized by various cell types, playing a key role in modulating survival and effector immune responses (Elvington et al., 2017; Kulkarni et al., 2019). Thus, local C3 activity plays an important role in modulating mucosal immune responses (Kulkarni et al., 2024).

‘Trained immunity’ refers to cells such as innate immune cells (i.e., monocytes and macrophages) exhibiting robust, enhanced inflammatory responses upon secondary stimulation after a prior insult (Quintin et al., 2012; Saeed et al., 2014). This form of ‘memory’ is considered broadly antigen nonspecific, yet lasts several months after the initial stimulus (Netea et al., 2020). Underlying the induction of trained immunity are epigenetic and metabolic reconfigurations occurring after an initial stimulus, which prime transcriptional machinery for rapid activation after engaging secondary insults (Bekkering et al., 2014; Fanucchi et al., 2021). For instance, the bacterial and fungal cell wall component 1,3-D-β-glucan binds the dectin-1 pattern recognition receptor, which induces metabolic changes favoring persistent glycolytic activity (Cheng et al., 2014; Earhart et al., 2023). This, in turn, promotes shifts in epigenetic modifications such as histone acetylation and methylation favoring strong proinflammatory gene expression upon restimulation, and can be long-lasting (Fok et al., 2018; Tercan et al., 2021). While knowledge of trained immunity is primarily systemic, there is growing evidence for site-specific effects such as in alveolar macrophages (AMs) (Chakraborty et al., 2023; Zahalka et al., 2022). Little is currently known about how the complement system affects trained immunity. Here, we sought to investigate whether the C3 protein – a key component of immune responses at mucosal sites such as the respiratory system – affects trained immunity in AMs and determine whether any effect is mediated by C3aR activity.

## Results

**Figure 1.**
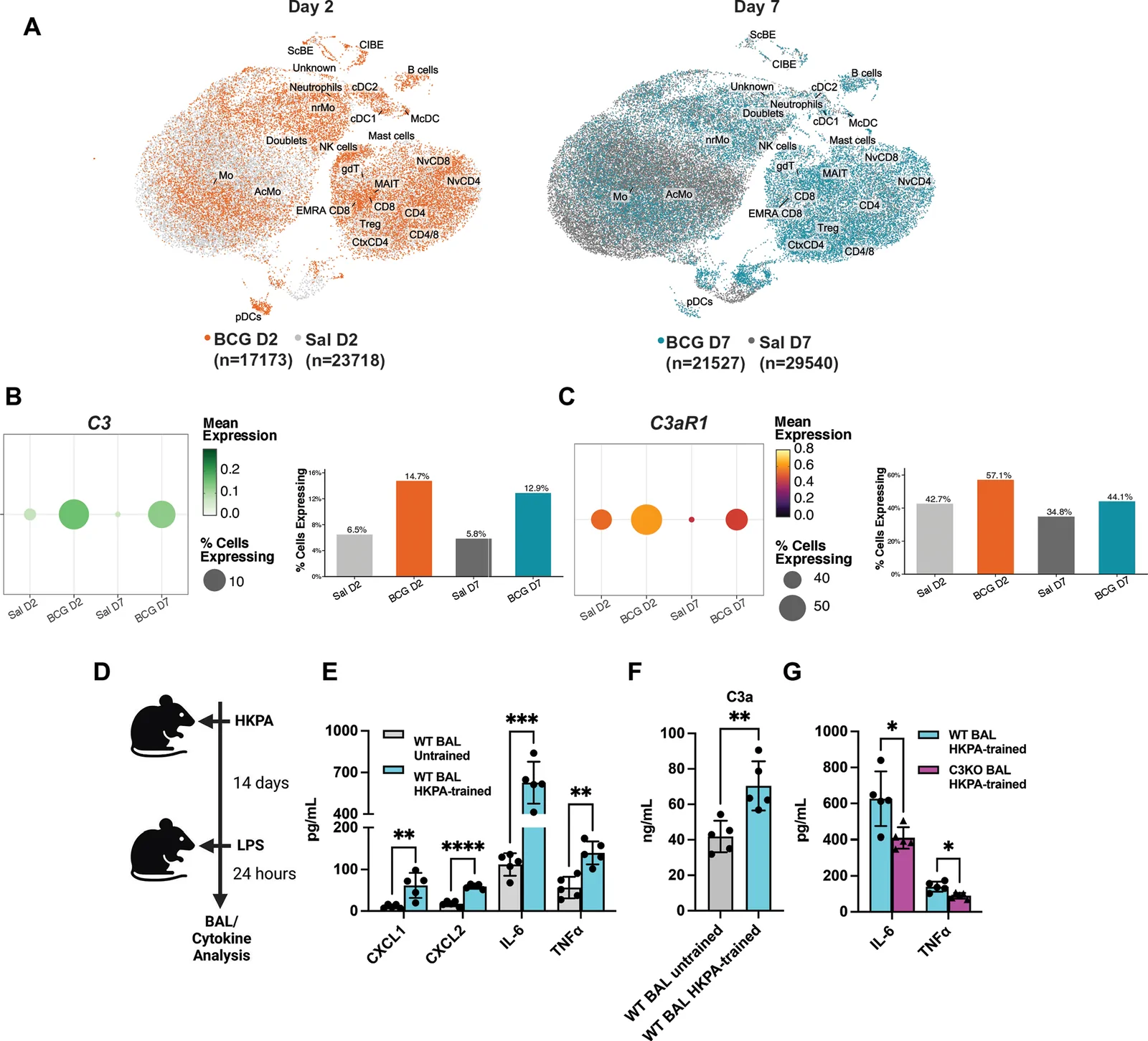
C3 deficiency predisposes to impaired pulmonary trained immunity. (**A**) UMAP (Uniform Manifold Approximation and Projection) plots showing the identity of each cell cluster in human bronchoalveolar lavage (BAL) from BCG- and saline (Sal)-treated donors at Day 2 (BCG *n* = 17,173; Sal *n* = 23,718) and Day 7 (BCG *n* = 21,527; Sal *n* = 29,540) post-vaccination. *N* = 3 volunteers in each group. Cell clusters include: macrophage (Mo), activated macrophage (AcMo), non-resident macrophage (nrMo), NK cells, γδ T cells, plasmacytoid DC (pDC), conventional DC (cDC1, cDC2), migratory DC (McDC), B cells, MAIT cells, CD4, CD8, CD4/8, Cytotoxic-like (Ctx), terminally differentiated effector memory CD45RA-re-expressing CD8 T cells (EMRA CD8), naïve T cells (NvCD4, NvCD8), T regulatory cells (Treg), neutrophils, mast cells, doublets, ciliated bronchial epithelial cells (CIBE), secretory bronchial epithelial cells (ScBE), and unidentified cells (Unknown). Data from Marshall et al., 2025. (**B**) Dot plot and bar chart showing *C3* expression across all human alveolar macrophages (Mφ; Mo, AcMo, nrMo pooled) across conditions. Dot size reflects the percentage of cells expressing C3; dot color reflects mean normalized expression. Bar chart shows percentage of C3-expressing Mφ per condition. (**C**) As in (**B**), for *C3AR1*. BCG vaccination increases the proportion of C3AR1-expressing alveolar macrophages at both timepoints relative to saline controls. (**D**) Schematic representing the training of mice via the intranasal route with heat-killed *Pseudomonas aeruginosa* (HKPA) and subsequent restimulation with lipopolysaccharide (LPS), followed by BAL and cytokine analysis. Created with BioRender. (**E**) WT untrained compared against WT-trained BAL levels of CXCL1, CXCL2, IL-6, and TNFα. (**F**) Comparison of BAL C3a levels, similar to (**E**). (**G**) WT-trained versus C3-deficient (C3KO)-trained BAL concentrations of IL-6 and TNFα. WT-trained levels derived from (**E**) for comparison with C3KO-trained mice. Data were compared with two-sided unpaired *t*-tests with (**E, G**) or without (**F**) Holm–Šidák correction for multiple hypothesis testing. Each point represents a measurement from one mouse with at least *n* = 4 in each group, mean ± SD shown. *p < 0.05, **p < 0.01, ***p < 0.001.

To assess the relevance of C3 in response to a training stimulus, we interrogated a publicly available dataset of BAL specimens from human volunteers who underwent aerosolized Bacillus Calmette Guérin (BCG) administration (Marshall et al., 2025). Both the mean expression as well as the percentage of AMs expressing *C3* and *C3aR1* increased at Day 2 post-BCG exposure and remained elevated at Day 7, as compared to their expression in AMs of volunteers who received saline (Figure 1A–C). *C3* expression was highest in the activated AM subset (Figure 1—figure supplement 1A–C). To investigate the role of C3 in pulmonary immune cell-trained immunity, we inoculated C57BL/6J wild-type (WT) and B6.129S4-*C3^tm1Crr^*/J C3 knockout (C3KO) mice intranasally with heat-killed *Pseudomonas aeruginosa* (HKPA) for training or vehicle control (PBS, untrained). After 14 days, lipopolysaccharide (LPS) from *Escherichia coli* was also administered intranasally to both mouse strains to induce secondary stimulation for 24 hr, followed by euthanasia and BAL as previously described (Figure 1D; Sahu et al., 2023). BAL proinflammatory chemokines and cytokines (CXCL1, CXCL2, IL-6, and TNFα) were quantified using ELISAs. Additionally, C3a, which is generated when C3 is activated and cleaved, was also measured using an ELISA specific to its neo-epitope. CXCL1, CXCL2, IL-6, and TNFα were all significantly elevated in trained versus untrained WT BAL (Figure 1E). Levels of C3a were increased in trained versus untrained WT BAL, indicating enhanced C3 activation is part of the trained immune response (Figure 1F). The enhancement in BAL IL-6 and TNFα with training was blunted in C3-deficient mice compared to WT mice (Figure 1G). Of note, no difference was observed in BAL protein, neutrophils, and cytokines (i.e., CXCL1, TNFα) at Day 14 (prior to LPS exposure) in C3-deficient compared to wild-type mice, regardless of prior HKPA (training) exposure (Figure 1—figure supplement 1D–G), suggesting the resolution of acute inflammation prior to the second stimulus. These results suggest C3 is required for trained immunity in vivo, and that the observed responses to the secondary challenge are not confounded by persistent inflammation from the initial exposure.

**Figure 1—figure supplement 1.**
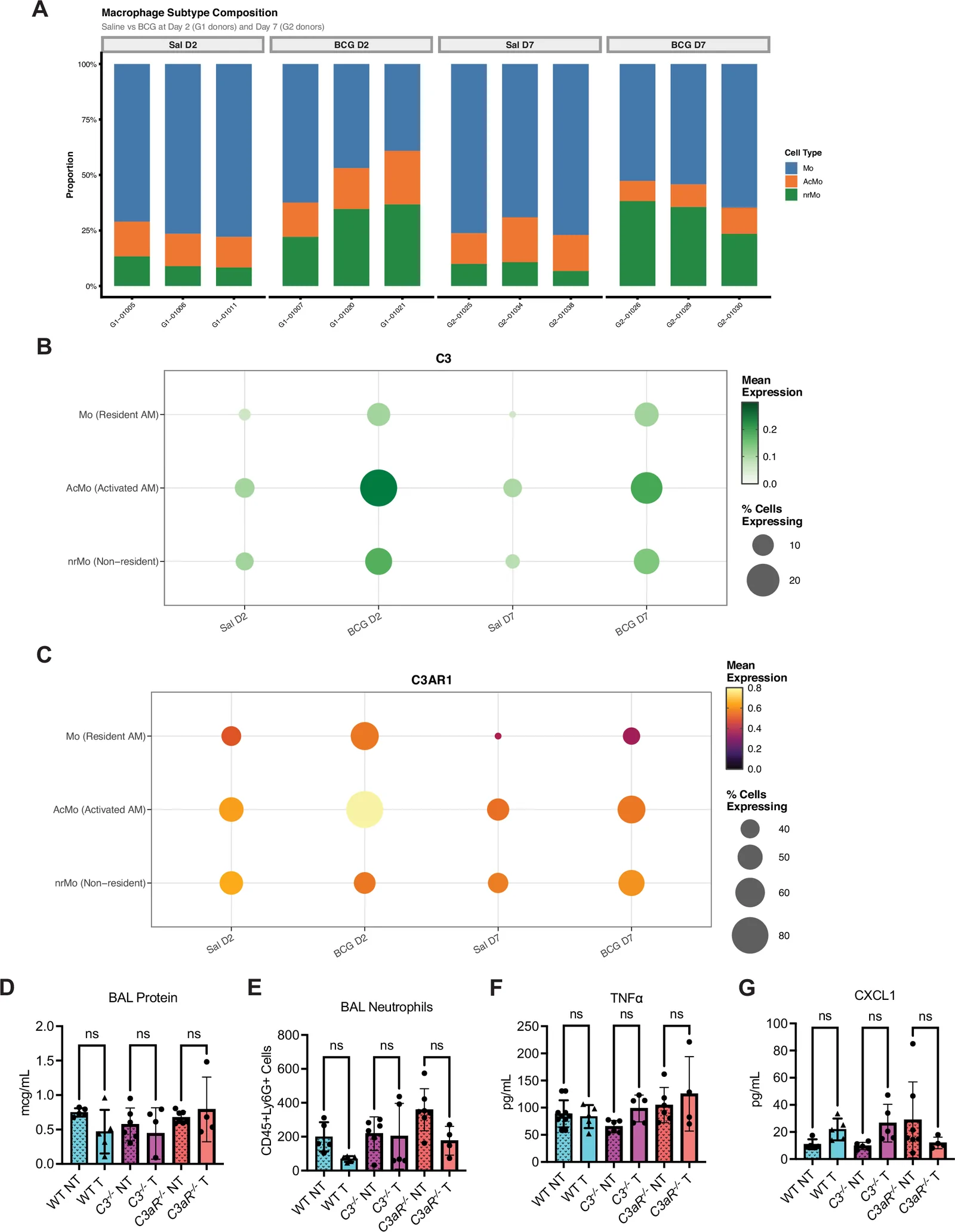
C3 deficiency predisposes to impaired pulmonary trained immunity. (**A**) Stacked bar charts showing macrophage subtype composition per donor across conditions. Proportions of resident alveolar macrophages (Mo), activated macrophages (AcMo), and non-resident monocyte-derived macrophages (nrMo) are shown for saline (Sal) and BCG-treated donors at Day 2 (G1 donors) and Day 7 (G2 donors). Data from Marshall et al. that included 91,958 cells from 12 samples [Day 2: Saline (3); BCG (3), Day 7: Saline (3); BCG (3)] consisting of BCG and saline-treated individuals at Days 2 and 7 after inhalation. (**B**) Dot plots showing *C3* expression across human alveolar macrophage subtypes (Mo, AcMo, nrMo) in saline and BCG-treated donors at Days 2 and 7. Dot size reflects the percentage of cells expressing *C3*; dot color reflects mean normalized expression. Data from Marshall et al. (**C**) As in (**B**), for *C3AR1*. Comparison of protein (**D**), neutrophils (**E**), TNFα (**F**), and CXCL1 (**G**) in the BAL of wild-type (WT), C3-deficient (C3^−/−^), and C3aR-deficient (C3aR^−/−^) mice at 14 days after training via the intranasal route with heat-killed *Pseudomonas aeruginosa* (HKPA). Data were compared with two-sided unpaired *t*-tests. Each point represents a measurement from one mouse with at least *n* = 4 in each group, mean ± SD shown. *p < 0.05, ns, non-significant.

**Figure 2.**
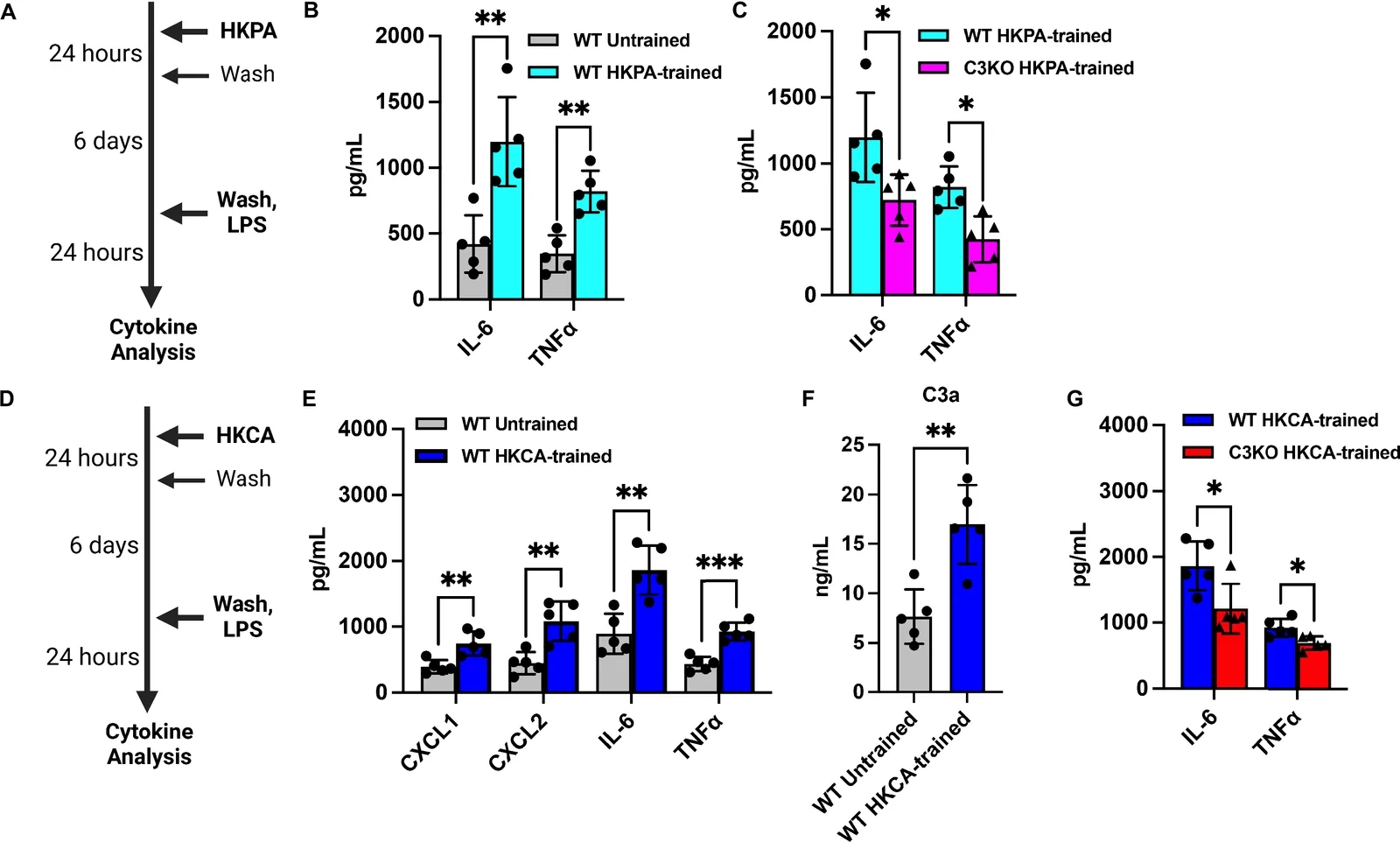
C3 deficiency results in impaired trained immune responses in ex vivo alveolar macrophages (AMs). (**A**) Schematic representing in vitro training of AMs with HKPA, with later stimulation by lipopolysaccharide (LPS) and subsequent cytokine analysis of the supernatants. Created with BioRender. Effects of HKPA-induced training in vitro on IL-6 and TNFα in supernatant from (**B**) WT AM, and (**C**) their comparison with C3KO-trained AMs. (**D**) Schematic representing in vitro training of AMs with heat-killed *Candida albicans* (HKCA), with subsequent restimulation by LPS and cytokine analysis of the supernatants. Created with BioRender. (**E**) Effects of HKCA-induced training in vitro on CXCL1, CXCL2, IL-6, and TNFα in supernatant from WT AM. (**F**) Comparison of C3a levels post-HKCA training, similar to (**B**). (**G**) Comparison of IL-6 and TNFα post-HKCA training in WT versus C3KO AMs. WT-trained levels derived from (**D**) for comparison with C3KO-trained AMs. Data were compared with two-sided unpaired *t*-tests with (**B, C, E, G**) or without (**F**) Holm–Šidák correction for multiple hypothesis testing. Each point is a technical replicate made by pooling AMs from at least *n* = 4 mice in each group, with mean ± SD shown, and each experiment was repeated twice. *p < 0.05, **p < 0.01, ***p < 0.001.

To examine how C3 affects trained immunity specifically in tissue-resident phagocytes, we set up an ex vivo culture system using primary AMs from C3-deficient and wild-type mice (Gorki et al., 2022). Like the in vivo experiments (Figure 1), we used HKPA to induce trained immunity in cultured AMs, followed days later by LPS challenge and analysis of cytokine secretion in the presence or absence of C3 deficiency (Figure 2A). Supporting our in vivo results, training by HKPA was sufficient to augment LPS-induced proinflammatory cytokine production from primary AMs (Figure 2B). However, C3-deficient AMs demonstrated blunted LPS-induced proinflammatory cytokine production post-training, as measured by IL-6 and TNFα secretion (Figure 2C) as well as impaired phagocytosis and reactive oxygen species (ROS) production (Figure 2—figure supplement 1A–C). Employing heat-killed *Candida albicans* (HKCA) in place of HKPA produced similar results (Figure 2D–G). To assess if the in vivo training specifically influenced AMs, we also trained the mice in vivo using HKPA using PBS as a control for 14 days, then harvested AM, and provided the second stimulus (LPS) ex vivo. In vivo trained AM from C3-deficient mice demonstrated a blunted cytokine response to LPS ex vivo compared to AM from wild-type mice (Figure 2—figure supplement 1D). These data suggest a specific role for C3 in AM immune training, consistent with our in vivo observations.

**Figure 2—figure supplement 1.**
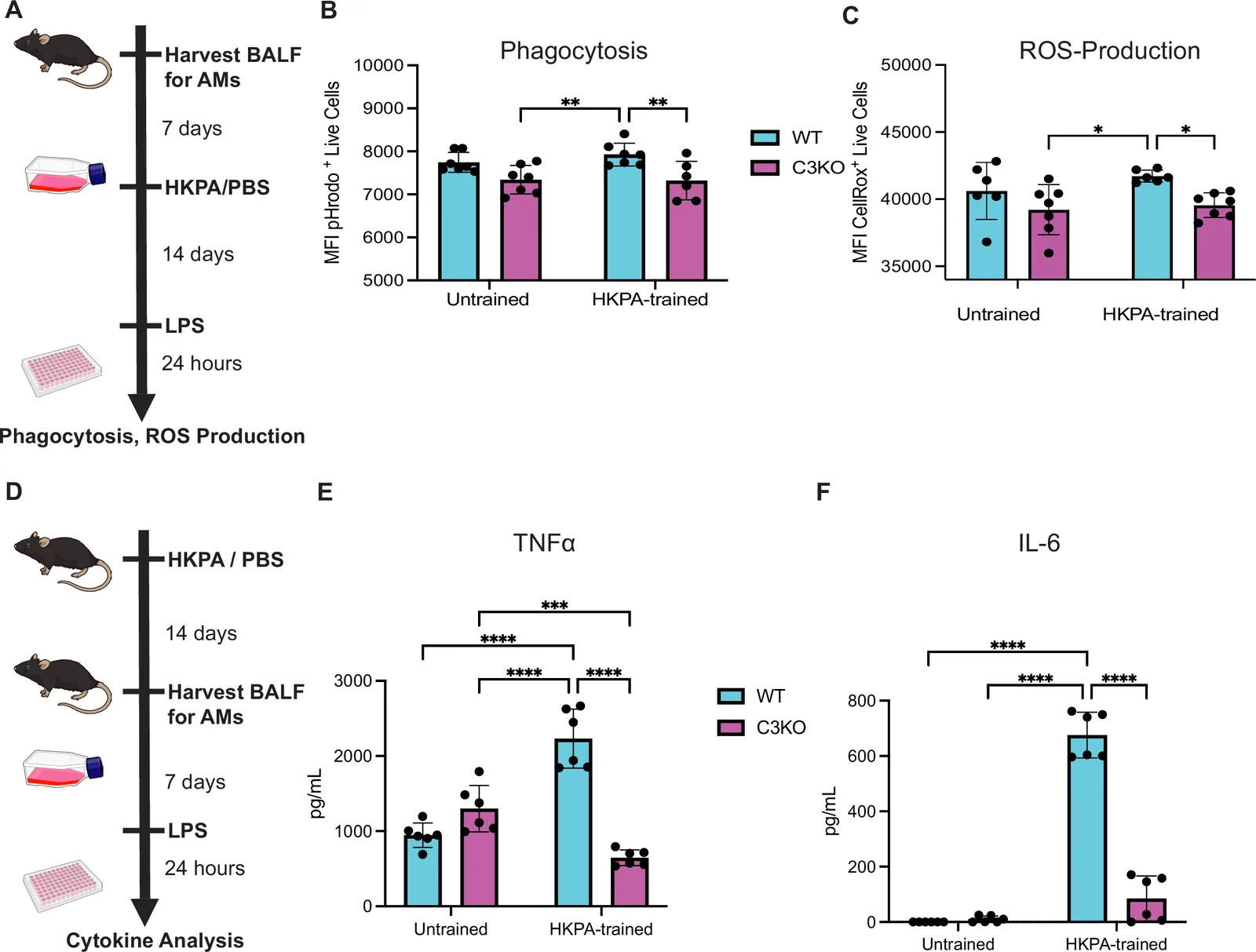
C3 deficiency results in impaired trained immune responses in lipopolysaccharide (LPS)-stimulated alveolar macrophages (AMs). (**A**) Schematic representing in vitro training of AMs with HKPA, followed by ex vivo stimulation using LPS and analyses of the cells. (**B**) Phagocytic capacity of in vitro HKPA-trained or untrained mouse AMs from WT and C3KO mice, measured by mean fluorescence intensity (MFI) of pHrodo-labeled *E. coli* bioparticles in live cells. (**C**) Reactive oxygen species (ROS) production in vitro in HKPA-trained or untrained mouse AMs from WT and C3KO mice, measured by CellRox MFI in live cells. (**D**) Schematic representing in vivo training of mouse lungs with HKPA, followed by ex vivo stimulation of harvested AM using LPS and subsequent cytokine analysis of the supernatants. Supernatants were collected at 24 hr post-LPS stimulation for cytokine analysis. TNFα and IL-6 concentrations in supernatants from untrained and HKPA-trained WT and C3KO AMs are shown. Data shown as mean ± SD; each point represents one biological replicate. Statistical comparisons by one-way ANOVA with multiple comparisons correction. *p < 0.05, **p < 0.01, ***p < 0.001, ****p < 0.0001. Illustrations in this figure from NIAID NIH BioArt Source Mouse (bioart.niaid.nih.gov/bioart/279), Flask (bioart.niaid.nih.gov/bioart/303), and 96 Well Plate (bioart.niaid.nih.gov/bioart/7).

**Figure 3.**
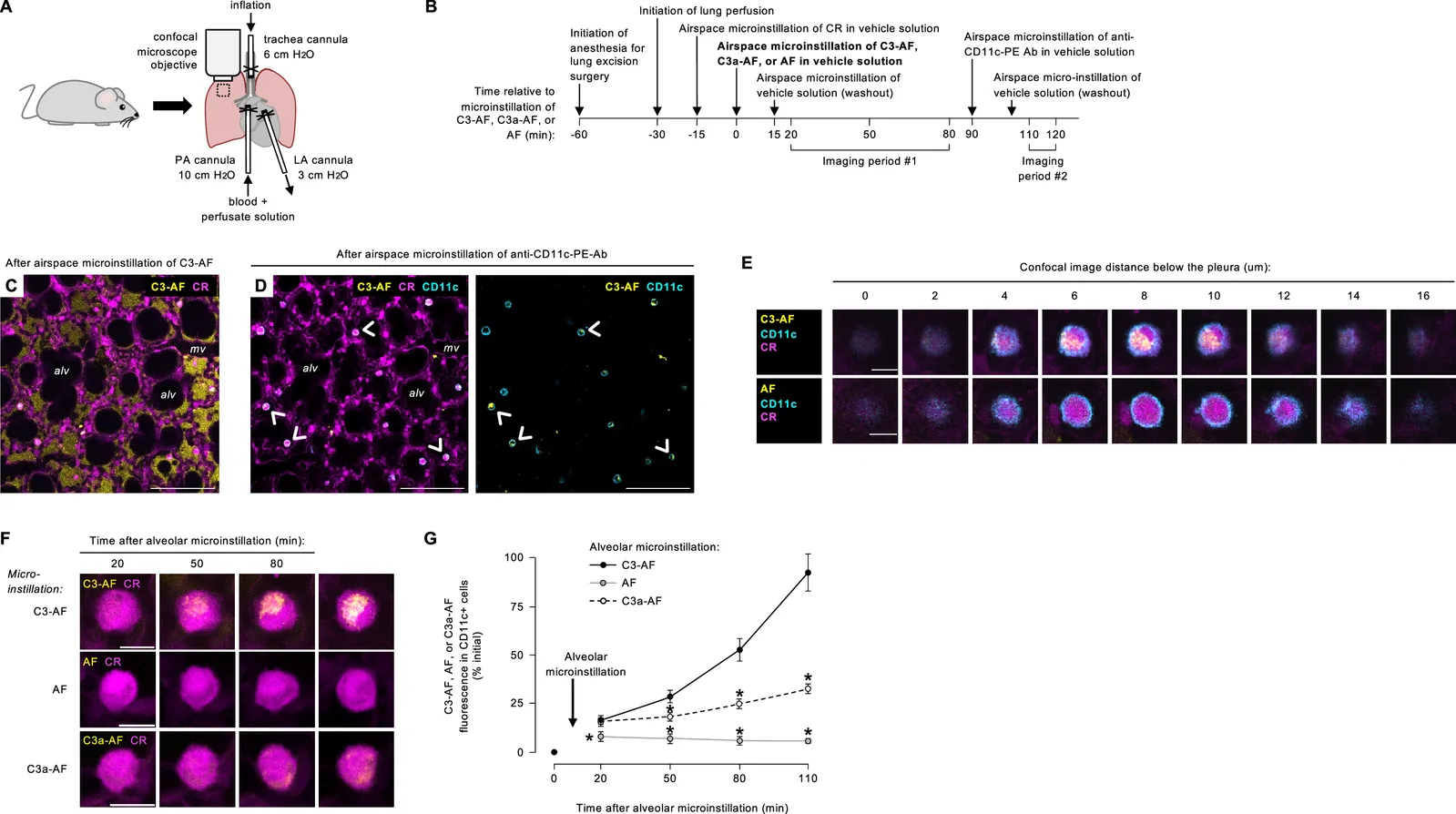
Alveolar macrophages take up C3 from airspaces of live alveoli in situ. (**A–G**) Cartoons in A, B show the experimental design of the confocal imaging studies shown in C−G. As indicated in A, B, we microinstilled alveolar airspaces of live, intact, perfused mouse lungs sequentially with: cell-permeant calcein red-orange dye (*CR*); Alexa Fluor 647 (*AF*), AF-tagged C3 (C3–AF), or AF-tagged C3a (*C3a-AF*); and phycoerythrin (*PE*)-tagged anti-CD11c Ab. The confocal image in C shows C3-AF fluorescence (*yellow*) in alveolar airspaces and CR fluorescence (*magenta*) in airspace-facing cells, including the alveolar epithelium and alveolar macrophages. *alv,* example airspace; *mv,* microvessel. Confocal images in D show the same alveoli, but CD11c fluorescence (*cyan*) now marks CD11c+ cells. *Arrowheads* point out example CD11c+ cells with intracellular C3-AF fluorescence. High power confocal images of CD11c+ cells (**E–F**) and group data (**G**) show C3-AF accumulated in cytosols of CD11c+ cells over time. In G, *circles* indicate mean ± SEM fluorescence in all of the CD11c+ cells present in imaging fields of at least 30 alveoli; *n* = 4 microinstillations in 2 lungs per group; *p < 0.05 versus C3-AF by ANOVA with post hoc Tukey testing. C3-AF, C3a-AF, and AF fluorescence in airspaces was normalized to C3-AF, C3a-AF, and AF fluorescence in glass micropipettes. Scale bars: 100 (**C, D**) and 10 (**E, F**) µm.

In addition to synthesizing C3, multiple cell types internalize C3 as C3(H_2_O) in vitro, which promotes cell survival and effector responses (Elvington et al., 2017; Kulkarni et al., 2019). To assess if C3 uptake by AMs can overcome the defect in responses from C3-deficient AMs, we first interrogated if mouse AMs internalize C3 in vivo. Labeled C3a was separately used as a control. Real-time confocal microscopy of live, intact mouse alveoli (Figure 3A, B) revealed that CD11c^+^ AMs internalize fluorescently labeled C3 rapidly after alveolar microinstillation, as compared to C3a (Figure 3C–G). Incubation of human precision-cut lung slices with labeled C3 revealed that human AM also internalize C3 in situ, thereby validating our observations from the intact mouse lung using human lung tissues (Figure 3—figure supplement 1).

**Figure 3—figure supplement 1.**
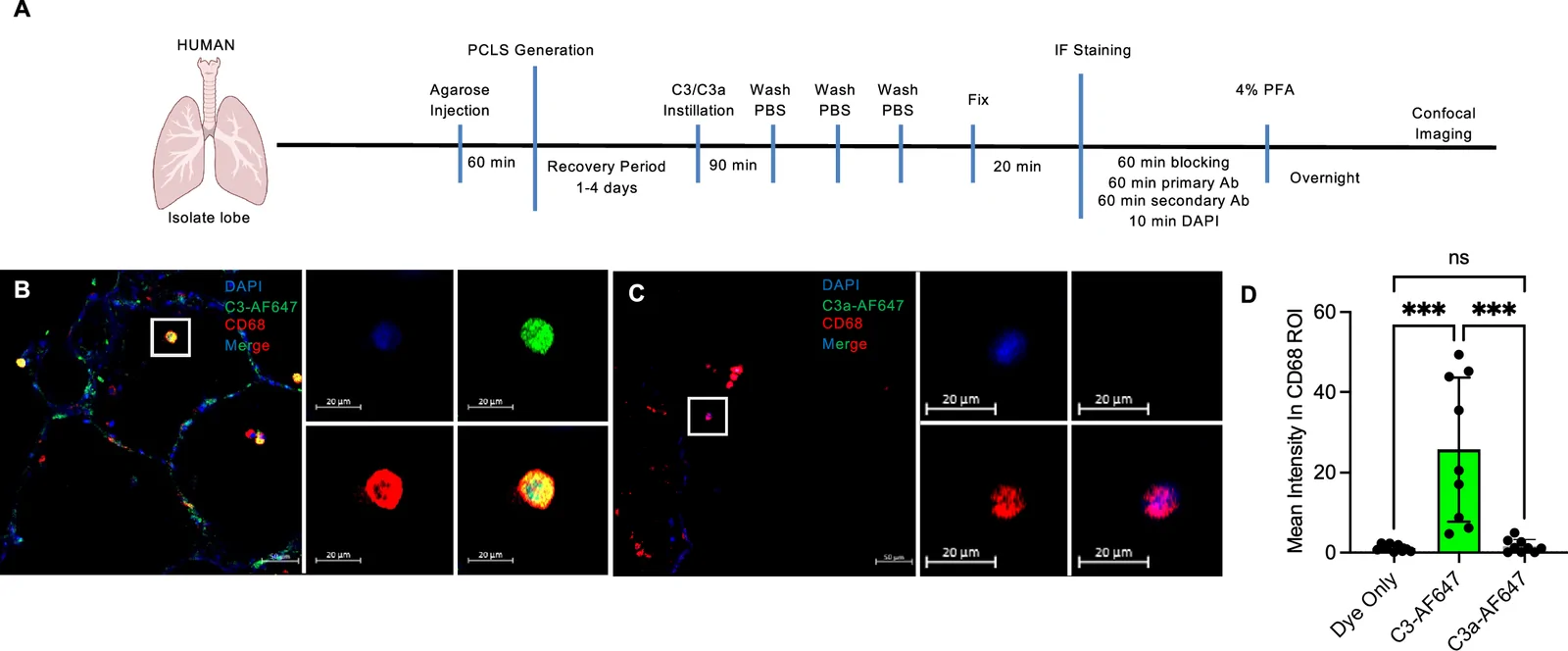
C3 uptake in human precision-cut lung slices (hPCLS). (**A**) Schematic for obtaining human PCLS. Schematic obtained from NIAID Visual & Medical Arts. (10/7/2024). Human Lungs. NIAID NIH BIOART Source. bioart.niaid.nih.gov/bioart/231. (**B**) Representative image showing C3-AF647 (green) incubated with CD68^+^ macrophages (red). Inset shows individual colors. DAPI: blue. (**C**) Representative image showing C3a-AF647 (green) incubated with CD68^+^ macrophages (red). Inset shows individual colors. DAPI: blue. (**D**) Comparison of individual data points from *n* = 3 hPCLS, 3 technical replicates per donor. *Y*-axis represents the mean fluorescence within the region of interest (ROI) defined by CD68. Data shown as mean ± SD. Statistical comparisons by one-way ANOVA with multiple comparisons correction. ***p < 0.001.

**Figure 4.**
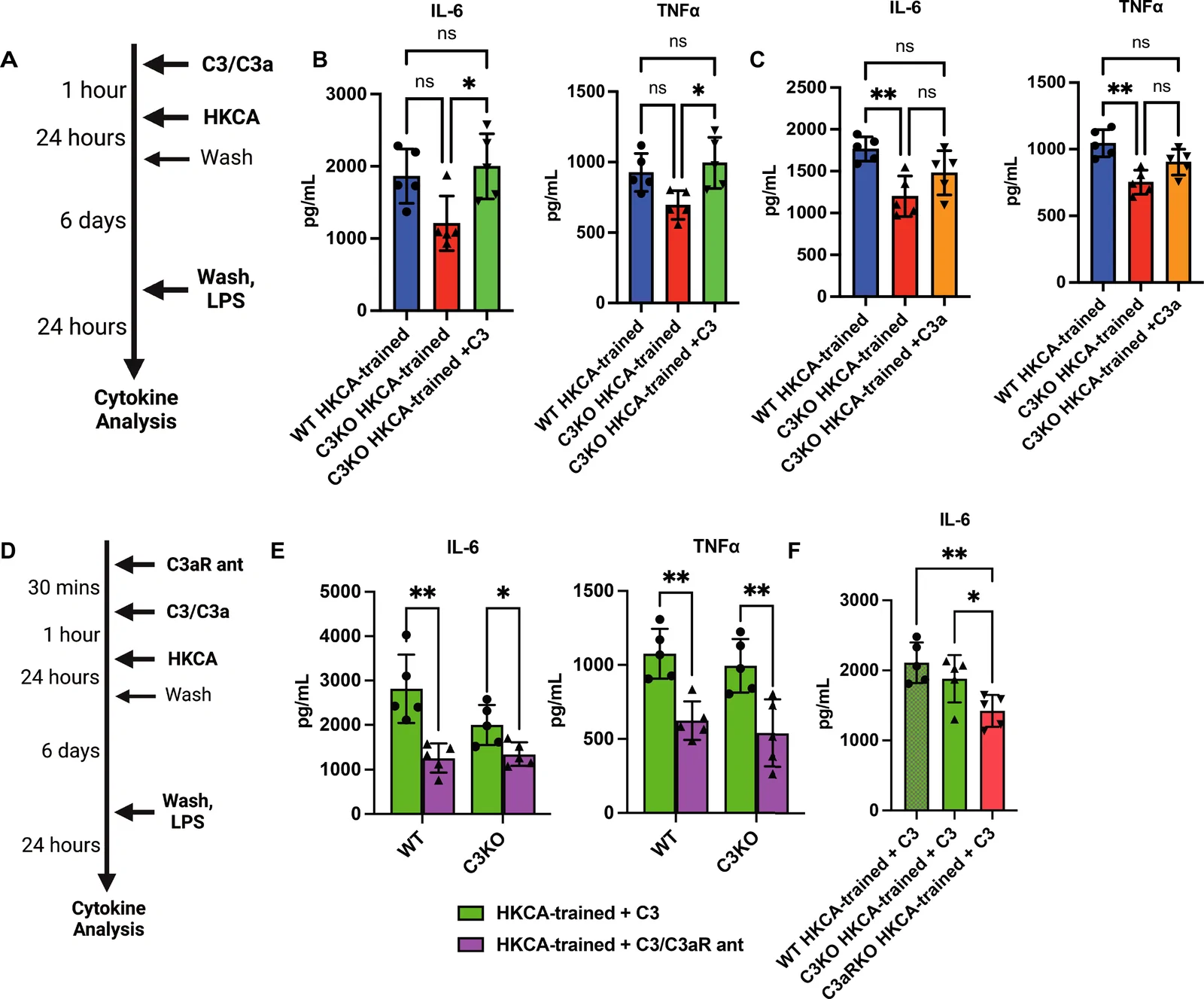
C3 uptake enhances trained immune responses in ex vivo alveolar macrophages (AMs) via the C3a receptor (C3aR). (**A**) Schematic representing in vitro training of AMs with heat-killed *Candida albicans* (HKCA), with pre-treatment of C3 or C3a prior to induction of training, and later stimulation by lipopolysaccharide (LPS) and subsequent cytokine analysis of the supernatants. Created with BioRender. (**B**) Effects of adding C3 prior to training on IL-6 and TNFα levels from C3KO AMs and their comparison with WT-trained AMs. (**C**) Effects of adding C3a prior to training, similar to (**B**). (**D**) Schematic representing addition of the C3aR antagonist prior to C3 treatment and in vitro training of AMs with HKCA, with later stimulation by LPS and subsequent cytokine analysis of the supernatants. Created with BioRender. (**E**) Effects of C3aR antagonism on IL-6 and TNFα levels from trained WT and C3KO AMs treated with exogenous C3. (**F**) Comparison of IL-6 levels post-HKCA-training in C3aR-deficient (C3aRKO), C3KO, and WT AMs treated with exogenous C3. Data were compared using one-way ANOVA with Dunnett’s post hoc tests (**B, C, F**) or two-sided unpaired *t*-testing with Holm–Šidák correction for multiple testing (**E**). Each point is a technical replicate made by pooling AMs from at least *n* = 4 mice in each group, with mean ± SD shown, and each experiment was repeated twice. *p < 0.05, **p < 0.01.

To mechanistically validate the role of C3 uptake in the training of AMs, we cultured C3-deficient AMs with exogenous C3 at a dose that leads to the cellular uptake of C3(H_2_O) (15 µg/ml, Figure 4A; Elvington et al., 2017; Kulkarni et al., 2019). As a control, we incubated cells with C3a, which is not internalized rapidly (Mogilenko et al., 2022). As measured by IL-6 and TNFα after a secondary stimulus, exogenous C3 protein restored the trained responses by AM to WT levels (Figure 4B). In contrast, these responses were not restored to WT levels by exogenous C3a, which stays outside the cell (Figure 4C).

Upon internalization, C3 is cleaved to C3a (Elvington et al., 2017). Intracellular C3a binds to C3aR and polarizes cytokine production in CD4^+^ T cells (Liszewski et al., 2013). To investigate whether a similar pathway could be important to C3-mediated trained immunity, we pre-treated AMs with a cell-permeable C3aR antagonist (SB290157) before immune training (Figure 4D). C3aR antagonism blunted LPS-induced cytokine production in both WT and C3-deficient AMs that had previously been rescued with exogenous C3 (Figure 4E). Importantly, genetic C3aR deficiency phenocopied the blunting of cytokine production seen in C3-deficient cells. Specifically, this defect in cytokine production post-training could not be rescued by exogenous C3 in C3aR-deficient AMs (Figure 4F). Altogether, these findings support a causal role for C3 in AM trained immunity via the C3aR.

**Figure 5.**
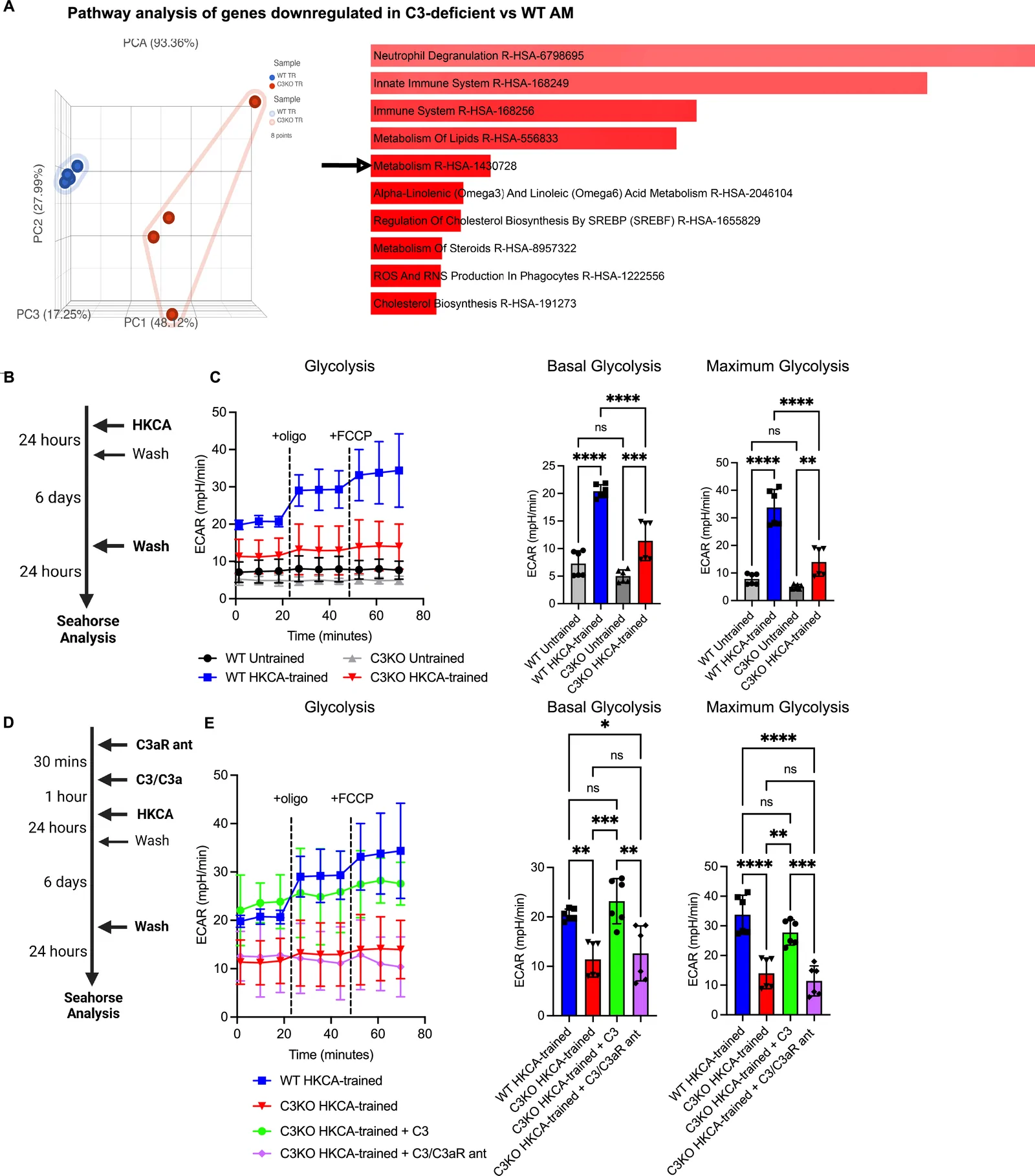
C3–C3aR axis is required for glycolysis as a part of trained immune responses in alveolar macrophages (AMs). (**A**) Principal component analysis (PCA, left) and EnrichR analysis of 391 genes (right, Supplementary file 1) downregulated in heat-killed *Candida albicans* (HKCA)-trained C3KO versus WT AMs by filtering genes (FDR step up ≤0.05). Arrow shows metabolism gene set in EnrichR; bars ranked by p-value. (**B**) Schematic representing in vitro training of AMs with HKCA. Created with BioRender. (**C**) Extracellular acidification rate (ECAR) from Seahorse analysis representing full glycolytic activity, and basal and maximum glycolysis in untrained and HKCA-trained WT and C3KO AMs. (**D**) Schematic representing addition of the C3aR antagonist (SB290157) prior to C3 treatment and in vitro training of AMs with HKCA. Created with BioRender. (**E**) Seahorse analysis in the presence and absence of exogenous C3 supplementation and C3aR antagonism. Each point is a technical replicate of pooled AMs from at least *n* = 4 mice in each group, with mean ± SD shown. *p < 0.05, **p < 0.01, ***p < 0.001, ****p < 0.0001 when analyzed using one-way ANOVA with Dunnett’s post hoc tests.

To explore transcriptomic changes relevant to how C3 influences trained immune responses, we performed bulk RNA-seq comparing trained WT to trained C3KO AMs. C3 deficiency led to differential expression of not only innate immune genes but also several genes involved in metabolism post-training, including those relevant to glycolysis such as *gnpda1* and *aldoc* (Figure 5A, Supplementary file 1). These metabolism-linked genes are significant because prior studies show that enhanced glycolytic metabolism is critical for trained immunity (Cheng et al., 2014). Therefore, we investigated whether the absence of C3 affects glycolytic flux in AMs (Figure 5B). As measured by extracellular acidification rate, untrained C3-deficient AMs had comparable basal and maximum glycolytic flux compared to WT but failed to augment glycolysis upon training with HKCA (Figure 5C). We next tested the ability of exogenous C3 to rescue the induction of training-associated glycolysis in C3-deficient AMs (Figure 5D). Exogenous C3 returned glycolysis induction to the level of trained WT cells, suggesting intracellular processing of C3 is important for this effect (Figure 5E). Like cytokines (Figure 4), inhibiting C3aR prevented exogenous C3 from rescuing glycolysis induction during immune training (Figure 5E).

## Discussion

Our data suggest C3 is upregulated after an initial inhalational exposure and is required for effective reprogramming of AMs. In its absence, AMs demonstrate blunted immune and metabolic responses to a subsequent stimulus, which are rescued by exogenous C3 and are C3aR-dependent. Of note, an intracellular C3aR has been reported by several groups (Liszewski et al., 2013; Quell et al., 2017; Zha et al., 2019). We show that C3 is rapidly internalized by AMs, similar to other immune and non-immune cell types (Elvington et al., 2017; Ishii et al., 2021; Kulkarni et al., 2019), whereas C3a in itself is not internalized. We propose that in AMs, the internalization of C3(H_2_O) provides an intracellular source of C3a (first suggested in Elvington et al., 2017), which potentially engages with the C3aR to result in reprogramming. While our data suggest that exogenously added C3a does not significantly affect AM reprogramming in our model system, we cannot rule out that other C3-mediated paracrine signaling mechanisms may also be at play. Regardless, our data indicate that C3 makes a mechanistic contribution to trained immunity in AMs and implicate engagement of the C3aR as part of the pathway. We also acknowledge that other immune cells such as dendritic cells and neutrophils, and non-immune cells such as epithelial cells, endothelial cells, and fibroblasts can also be involved in immune training (Bigot et al., 2025; Friščić et al., 2021; Moorlag et al., 2020).

Strengths of our study include in vitro and in vivo approaches to inducing trained immunity, orthogonal endpoints to quantify immune training (cytokine and ROS production, phagocytosis, and metabolic readouts), and mechanistic gain- and loss-of-function experimental approaches. Future work involves identifying the downstream effectors of C3 and C3aR in the AM training process and their connection to immunometabolism. We also need to establish whether C3a–C3aR-mediated AM reprogramming can lead to altered disease outcomes. Systemically administered β-glucan has been shown to induce peripheral trained immunity and aggravate lung injury (Prevel et al., 2025) similar to disease in models of periodontitis and arthritis (Haacke et al., 2025). However, training with β-glucan also reduces bleomycin-induced lung fibrosis (Kang et al., 2024a). Hence, we aim to optimize relevant intrapulmonary exposures to assess how trained immune responses are modulated by the C3a–C3aR axis, while also clarifying the extent to which central C3–C3aR-mediated training alters AM responses. The results may have clinical implications since trained immunity is linked to vaccine effectiveness (e.g., with respect to the Bacillus Calmette-Guérin vaccine) and overall immune resilience (Arts et al., 2018; Kleinnijenhuis et al., 2015). Moreover, humans with C3 deficiency demonstrate impaired responses to vaccination (Kim et al., 2018; Pekkarinen et al., 2015). It is tempting to speculate that local augmentation of C3 and/or C3aR activity could enhance vaccine effectiveness for hard-to-vaccinate pathogens like *Mycobacterium tuberculosis*.

## Materials and methods

### Experimental model details

#### Mice

All animal studies were conducted on an approved IACUC protocol. C57BL/6J (RRID:IMSR_JAX:000664, termed wild-type, or WT) and B6.129S4-*C3^tm1Crr^*/J (RRID:IMSR_JAX:003641, termed C3-deficient, or C3KO) mice were obtained from Jackson Laboratory (Bar Harbor, ME, USA). The mice were maintained as groups in our animal housing facility at Washington University in St. Louis School of Medicine with a 12-hr light–dark cycle, with temperatures between 22 and 23°C and humidity of 68–72%. C3aR-deficient (termed C3aRKO) mice were provided by Dr. Rick Wetsel at the University of Texas in Houston, were bred in-house and have been reported previously by us (Kildsgaard et al., 2000; Sahu et al., 2023).

### Experimental design

The ARRIVE guidelines were followed for reporting of in vivo experiments, and are reported throughout the Methods and Figure Legends. Female and male mice between the ages of 8 and 16 weeks of age were used in these experiments and were age-matched in each experiment. Sample size was determined based on prior studies on acute lung injury; a minimum of five mice were used from each genotype (Sahu et al., 2023). Pre-established exclusion criteria for mice included being pregnant or injured (i.e., unanticipated pre-existing wounds from co-housing). Each experiment was repeated at least twice, and both biological and technical replicates were included. No sample or data point from the analysis was omitted.

### In vivo trained immunity in mice

WT and C3KO mice were administered heat-killed *Pseudomonas aeruginosa* (HKPA, Pa57-15, 1 × 10^5^ CFU/mouse) (Sahu et al., 2023) or PBS vehicle control intranasally. After 14 days, LPS (*E. coli* O111:B4, Millipore Sigma, cat #: L4391, 10 µg/mouse) or PBS was also intranasally given to these mice. 24 hr later, mice were anesthetized with 1.25% tribromoethanol (125–250 mg/kg) via intraperitoneal injection followed by cervical dislocation for euthanasia. BAL was performed by inserting a 20Gx1” Surflo IV catheter into the trachea and instilling approx. 0.5 ml PBS + protease inhibitor (Halt Protease and Phosphatase Inhibitor Single-Use Cocktail (100X), Thermo, cat #: 78444) into the lungs. The fluid was then collected, followed by centrifugation at 3000 rpm for 3 min at 4°C for separating the cell pellet and supernatant, which was then stored at –20°C until later use.

### AM isolation and maintenance

Ex vivo AM were cultured as per a previously published protocol (Gorki et al., 2022; Zahalka et al., 2022). To obtain AM, BAL was performed at least 4 times per mouse, followed by centrifugation at 3000 rpm for 3 min at 4°C. The resulting cell pellet was resuspended in AM media (RPMI + 10% FBS [Biowest, cat #: S1620], 1% penicillin + streptomycin, 1 µM Rosiglitazone [Millipore Sigma, cat #: R2408], 10 ng/ml mouse TGF-β1 [BioLegend, cat #: 781804], and 30 ng/ml mouse GM-CSF [PeproTech, cat #: 315-03-100UG]), plated on 25 mm round dishes, and incubated at 37°C + 5% CO_2_ for 24 hr. Media was then removed, and cells were washed twice with warm PBS to remove unwanted cells and debris. AM media was added again, and cells allowed to grow for at least 7 days. After this, cells were washed again with warm PBS then incubated for 15–20 min with Accutase (Thermo Fisher, cat #: MT25058CI) to gently remove them from the plates. They were then centrifuged at 500 × *g* for 5 min at 4°C, resuspended in AM media, then placed in T25 flasks and incubated at 37°C + 5% CO_2_. Resulting AMs were passaged every 7 days for a maximum of 25 passages (Gorki et al., 2022).

### Induction of trained immune responses in AM in vitro

AMs were plated at 1 × 10^5^ cells/well in 96-well flat-bottom tissue culture-treated plates and incubated at 37°C + 5% CO_2_ for 30 min to 1 hr to promote adherence. Each well was then washed with PBS, followed by the addition of ‘trained’ AM media supplemented with HKPA or HKCA (1 × 10^4^ CFU HKPA or HKCA/well, Invitrogen, cat #: tlrl-hkca) or a matched volume of PBS as a vehicle control for “untrained” wells. Plates were incubated for 24 hr at 37°C + 5% CO_2_, washed with PBS, then rested for 6 days in AM media, followed by secondary stimulation with LPS (10 ng/ml, *E. coli* O111:B4, Millipore Sigma, cat #: L4391). After 24 hr, supernatants were collected and frozen at –20°C until later use.

### Exogenous treatment of AM with C3 and C3a

In order to investigate the effects of C3 uptake or C3a individually on training, either C3 (15 µg/ml, CompTech, cat #: M113) or C3a (10 µg/ml, CompTech, cat #: A118) were added to wells 1 hr prior to training of WT, C3KO, or C3aRKO AMs with HKCA. To examine whether C3/C3a affects training via the C3a receptor (C3aR), wells were supplemented with 200 nM of C3aR antagonist SB290157 (Millipore Sigma,cat #: SML1192) 30 min prior to either training with HKCA or addition of exogenous C3 or C3a followed by training 1 hr later.

### Quantification of chemokines and cytokines

Untrained and trained BAL and AM supernatants obtained as described above were thawed to room temperature from –20°C. Concentrations of CXCL1, CXCL2, IL-6, and TNFα, or also RAGE and total protein in BAL, were determined via competitive ELISA plates (R&D Systems Inc, Minneapolis, MN, USA) or Milliplex plates (Millipore Sigma, St. Louis, MO, USA) according to manufacturer’s instructions at 1:2 or 1:4 dilution. Sandwich ELISAs were then read on an EPOCH microplate reader via optical density measurement at 450 nm wavelength. Multiplex ELISA plates (Millipore Sigma, St. Louis, MO, USA) were read using a Bio-Rad Luminex 100 multiplex system.

### Quantification of C3a-neo

The protocol for measuring C3a-neo in the supernatant was adapted from a previously published protocol measuring it in the serum (Pagano et al., 2009). ELISA plates (96-well flat-bottom; #3855, Thermo Fisher Scientific) were coated with Mouse C3a Capture Antibody (1:250; 100 µl per well of PBS; Purified Rat Anti-Mouse C3a, Cat# 558250, BD Pharmingen) overnight at 4°C. After washing three times with a solution of 0.05% Tween 20 in PBS, the plate was blocked with 1% bovine serum albumin (BSA; #A7906, Sigma-Aldrich) at room temperature (RT) for 1 hr. Plates were washed again and samples diluted at 1:4 in 1% BSA/PBS solution were added (100 μl per well). Standard curve, made from purified mouse C3a (Purified Mouse C3a Protein, Cat# 558618 BD Pharmingen) was used from 50 to 3.125 ng/ml. Samples and purified protein were incubated at RT for 2 hr. The plates were subsequently washed three times and Mouse C3a Detection Antibody (1:1000; 100 µl per well in 1% BSA/PBS; Biotin Rat Anti-Mouse C3a, #558251, BD Pharmingen) was diluted in 1% BSA. After another three washes, samples were incubated with Streptavidin HRP-conjugated (100 µl per well; 1:200 dilution, #DY998, R&D Systems) for 30 min at RT. After three washes, TMB Color Substrate (#DY999, R&D Systems) was added at 100 μl per well and incubated at RT for 5 min. The reaction was stopped by addition of 1 M sulfuric acid (50 μl per well; #DY994, R&D Systems), and optical density was measured at 450 nm (Epoch Microplate Spectrophotometer, BioTek).

### Phagocytosis measurements

Following experimental treatments, WT and C3KO AMs plated at 1 × 10⁵ cells/well in 96-well flat-bottom tissue culture-treated plates were washed once with PBS, followed by the addition of pHrodo Green *E. coli* BioParticles (Life Technologies, #P35366) at 25 µg/ml in AM media (100 µl/well). Cells were then incubated at 37°C + 5% CO₂ for 30 min. After this, cells were washed with warm PBS and incubated for 15–20 min with Accutase, then centrifuged at 500 × *g* for 5 min at 4°C. For viability discrimination, cells were resuspended in Zombie Violet (BioLegend, #423113) at a 1:2400 dilution in PBS (100 µl/well) and incubated at 4°C in the dark for 15 min. Cells were then washed twice in 1% BSA/PBS and resuspended in 200 µl of 1% BSA/PBS for flow cytometric analysis. Samples were acquired on an Attune Xenith spectral flow cytometer (Thermo Fisher Scientific) operating in conventional mode, using the violet laser (405 nm) for Zombie Violet excitation and the yellow-green laser (561 nm) for pHrodo Green excitation. Phagocytic activity was quantified as the median fluorescence intensity (MFI) of pHrodo Green-positive cells within the live AM gate (Zombie Violet⁻). Data were analyzed using FlowJo v10 (BD Biosciences).

### Measurement of ROS production

Following experimental treatments, WT and C3KO AMs plated at 1 × 10⁵ cells/well in 96-well flat-bottom tissue culture-treated plates were washed once with PBS, followed by the addition of AM media containing CellROX Green (Thermo Fisher Scientific, #C10444) at a final concentration of 500 nM. Cells were incubated for 30 min at 37°C + 5% CO₂ in the dark. After this, cells were washed with warm PBS and incubated for 15–20 min with Accutase, then centrifuged at 500 × *g* for 5 min at 4°C. For viability discrimination, cells were resuspended in Zombie Violet (BioLegend, #423113) at a 1:2400 dilution in PBS (100 µl/well) and incubated at 4°C in the dark for 15 min. Cells were then washed twice in 1% BSA/PBS and resuspended in 200 µl of 1% BSA/PBS for flow cytometric analysis. Samples were acquired on Cytek Aurora Spectral flow cytometer (Cytek Bioscience) using a full spectrum unmixing mode. Spectral unmixing was performed using single-color controls and an autofluorescence reference collected from unstained AMs. ROS production was quantified as the MFI of CellROX Green within the live AM gate (Zombie Violet⁻). Data were unmixed using SpectroFlo (Cytek Biosciences) and analyzed for downstream gating using FlowJo v10 (BD Biosciences).

### Flow cytometric measurement of neutrophils

BAL fluid was collected as described above and centrifuged at 500 × *g* for 5 min at 4°C to pellet cells. To remove red blood cells, the cell pellet was resuspended in ACK lysis buffer (150 mM NH₄Cl, 10 mM KHCO₃, 0.1 mM Na₂-EDTA, pH 7.2–7.4) for 2 min at room temperature, then quenched with 1% BSA/PBS and centrifuged at 500 × *g* for 5 min at 4°C. For viability discrimination, cells were resuspended in Zombie Violet (BioLegend, #423113) at a 1:2400 dilution in 1% BSA/PBS and incubated at 4°C in the dark for 15 min. Cells were then washed twice in 1% BSA/PBS and stained with CD45-PE/Cyanine7 (BioLegend) and Ly6G-FITC (BioLegend) for 20 min at 4°C in the dark. Cells were washed twice in 1% BSA/PBS and resuspended in 200 µl of 1% BSA/PBS for acquisition. Samples were acquired on an Attune NXT flow cytometer (Thermo Fisher Scientific) operating in conventional mode, using the violet laser (405 nm) for Zombie Violet excitation, the blue laser (488 nm) for FITC excitation, and the yellow-green laser (561 nm) for PE/Cyanine7 excitation. Neutrophils were identified by sequential gating on Zombie Violet⁻ (live) CD45^+^ Ly6G^+^ cells. Data were analyzed using FlowJo v10 (BD Biosciences).

### Cell metabolism

To examine a possible mechanistic basis for differences in trained immune responses in WT, C3KO, and C3aRKO AMs, cells were plated at 1 × 10^5^/well of an XFe24-well cell culture plate (Agilent Technologies, Santa Clara, CA, USA) and trained in AM media with or without C3/C3a, or SB290157 and subsequently washed and rested as described above. After 6 days, cells were restimulated with LPS (10 ng/ml) for 24 hr, washed, then left in mitochondrial stress test buffer (Agilent XF DMEM media supplemented with 10 mM glucose, 2 mM glutamine, and 1 mM sodium pyruvate, pH 7.4) at 37°C with no CO_2_ for 1 hr. Glycolysis by means of extracellular acidification rate via lactate production in the supernatant was analyzed using an Agilent Seahorse XFe24 analyzer. Three timepoints were measured each of stable basal glycolysis; after addition of 2.5 µM oligomycin and following the injection of 2 µM FCCP for maximal glycolysis; and was performed according to manufacturer instructions.

### Real-time confocal microscopy of live, intact mouse alveoli

#### Animals

Mice were Swiss Webster, were purchased from Charles River Laboratories and Taconic Biosciences, weighing 25–40 g, and at the age of 6–12 weeks old.

#### Solutions

We purchased Ca^2+^- and Mg^2+^-containing DPBS and Ca^2+^- and Mg^2+^-free PBS from Corning. Isolated mouse lungs were perfused with HEPES-buffered solution of pH 7.4 and osmolality 333 mOsm/L and containing 150 mM Na^+^, 5 mM K^+^, 1 mM Ca^2+^, 1 mM Mg^2+^, 140 mM Cl^-^, 10 mM glucose, 4% dextran (70 kDa; Molecular Probes), and 1% FBS (Gemini Bio-Products). Fluorophores, reagents, and antibodies microinstilled into alveoli were dissolved or suspended in HEPES-buffered vehicle solution containing 150 mM Na^+^, 5 mM K^+^, 1 mM Ca^2+^, 1 mM Mg^2+^, 140 mM Cl^-^, and 10 mM glucose.

#### Reagents

Reagents were freshly constituted for experiments. We purchased calcein red-orange AM (10 μM) and PE-tagged anti-CD11c Ab from Thermo Fisher Scientific. Mouse C3 and C3a were purchased from Complement Technology, Inc, incubated with Alexa Fluor-NHS Ester Kit per protocol (Thermo Fisher Scientific), and stored at –80°C prior to use.

#### Preparation of isolated, perfused lungs for microinstillation and imaging

We anesthetized mice with inhaled isoflurane (4%) and intraperitoneal injections of ketamine (up to 100 mg/kg) and xylazine (up to 5 mg/kg), then gave intracardiac injections of heparin (50 units; Mylan) and exsanguinated the mice by cardiac puncture. We used our established methods (Hook et al., 2018; Tang et al., 2023) to cannulate the trachea, pulmonary artery, and left atrium of the heart, then excise the heart, lungs, and cannulas en bloc. The lungs were positioned to enable micropuncture and imaging of the diaphragmatic surface of the right middle lobe, right caudal lobe, or left lung. Then, we inflated the lungs with room air through the tracheal cannula and perfused the lungs through the pulmonary arterial and left atrial cannulas at 0.5–1.0 ml/min with autologous blood diluted in the lung perfusate solution (see ‘Solutions’) and warmed to 37°C. We used in-line pressure transducers (ADInstruments) to maintain constant airway pressure 6 cm H_2_O via a continuous positive airway pressure machine (Philips Respironics) and pulmonary artery and left atrial pressures 10 and 3 cm H_2_O, respectively, via a roller pump (Ismatec). Portions of the lung surface that were not used for micropuncture and imaging were covered with plastic wrap to prevent desiccation.

#### Alveolar microinstillation

We utilized hand-beveled glass micropipettes (Sutter Instruments) to micropuncture single alveoli under bright-field microscopy, as we have done previously (Hook et al., 2018; Tang et al., 2023). Micropunctured alveoli were instilled with fluorophores and reagents in solution, resulting in their spread from the micropunctured alveolus to neighboring alveoli. Microinstillations were performed in 1–3 alveoli bordering each imaging field.

#### Live lung imaging and analysis

Using our established methods previously (Hook et al., 2018; Tang et al., 2023), we viewed alveoli by confocal microscopy (LSM800; Zeiss) with a 20x water immersion objective (NA 1.0; Zeiss) and coverslip. We used bright-field microscopy to randomly select regions of 30–50 alveoli for microinstillation and imaging. All images were acquired as single images using Zen (v.2.6; Zeiss) and recorded as Z-sections. Analyzed images were 4–8 μm below the pleura. Optical thickness was 2 μm, and frame size was 512 × 512 pixels. We established laser, filter, pinhole, and detector settings at the beginning of each imaging experiment to optimize alveolar fluorescence and avoid fluorescence saturation, then maintained the settings for the duration of the experiment. We confirmed the absence of bleed-through between fluorescence emission channels. Images were analyzed using ImageJ (NIH; v.2.0.0-rc-69/1.52n). Linear adjustments of brightness and contrast were applied to individual color channels of entire images and equally to all experiment groups. We did not apply downstream processing or averaging.

#### Statistics

Statistics are indicated in figures and legends. We considered statistical significance at p < 0.05. Data were analyzed and figures were prepared using Microsoft Excel, StatPlus:mac Pro (AnalystSoft, Inc, Build 7.5.0.0/Core v7.6.11), and SigmaPlot (Systat, version 14.5).

#### Study approval

The Institutional Animal Care and Use Committee of the Icahn School of Medicine at Mount Sinai approved the animal procedures.

### Human precision-cut lung slices preparation

Deidentified human lungs were obtained from OneLegacy. The left lobe was filled with 2% LMP agarose (VWR, 75816-210) kept warm at 40°C. Once the agarose had congealed, the lobes were excised and placed in ice-cold PBS. Outer pleura lining was removed from the lung lobe, lobes were sliced into ¾-in. cross sections, an 8-mm biopsy punch was used to create samples for sectioning, and airways were avoided due to difficulty with slicing. Punched samples were kept in ice-cold PBS until slicing. Lobes were then embedded for slicing (VF-510-0Z, Precisionary Instruments) at 350 µm in 2% LMP agarose. Slices were placed immediately into cold recovery media and allowed to warm to room temperature before moving to a 37°C incubator. Recovery media was changed after 30 min, 2 hr, and overnight incubation. Precision-cut lung slice (PCLS) media was changed each day afterwards.

### PCLS viability

Culture media was collected each day after initial slicing to determine viability through Invitrogen CyQUANT LDH and G6PD Cytotoxicity Assays (Fisher Scientific, C20301). This data was matched alongside LIVE/DEAD Viability/Cytotoxicity Kit for mammalian cells (Thermo Fisher, L3224). Two fresh slices were used each day for this assay. They were first stained for 30 min in 1 ml of 10 µl of Calcein in 10 ml of dPBS; followed by an additional 30 min with 1 µl of Ethidium Homodimer added to each well. Slices were imaged on ECHO Revolve Microscope at 4x. Viability was also confirmed through the presence of ciliary beating in the airways.

### Protein uptake

C3, C3, and C3a (Complement Technology) were first conjugated with Alexa Fluor 647 NHS Ester (Succinimidyl Ester) (Thermo Fisher, A20006). The final concentration of protein was brought up to be 1 µg/µl. Two PCLS slices were loaded per mouse with 30 µg of C3 for 90 min at 37°C. An equivalent amount of dye was loaded into wells for each mouse as a control. After incubation, cellular activity was stopped by fixation in 4% paraformaldehyde for 20 min.

### Fluorescence staining

After fixation, slices were washed in PBS and blocked in 5% BSA for 1 hr at room temperature. Slices were then washed again in PBS and stained for CD68 (Abcam, ab955) in human lungs at 1:100 concentration, followed by washing 3x. Secondary staining was done at 1:200 concentration with Donkey anti-Mouse IgG (H+L) Highly Cross-Adsorbed Secondary Antibody, Alexa Fluor 647 (Thermo Fisher, A31571),, followed by washing 3x. Counterstaining was done with DAPI at 5 µl in 10 ml of PBS for 10 min. Slices were then kept in 4% PFA overnight before imaging the next day on Zeiss LSM 880 Confocal Microscope. Z-stacks were taken for each slice; boundaries were determined by the presence and absence of DAPI.

### Fluorescence quantification

Quantification was done by a prewritten macro for ImageJ to reduce human error in repetitive processing. Each z-stack was converted to a png, each png was measured for green channel (C3/C3a) intensity. ROI was formed by thresholding on CD68 staining and masking (Gaussian Blur sigma = 2.5; thresholding 60–255; analyze particles size = 400–6000, circularity = 0.1–1.00, fill holes). Stacks returning no particles were excluded from analysis. Mean intensity found within ROI was then graphed on Prism.

### Single-cell RNA-sequencing

Data from BAL cells was downloaded from the Gene Expression Omnibus (accession number GSE282132). The dataset included 91,958 cells from 12 samples [Day 2: Saline (3); BCG (3), Day 7: Saline (3); BCG (3)] consisting of BCG and saline-treated individuals at Days 2 and 7 after inhalation. The processed H5AD file was imported into R using the Zellkonverter package (v1.16.0). Pre-computed dimensionality reduction results, including Harmony-corrected PCA and UMAP embeddings generated by the original authors (Marshall et al., 2025) using harmonypy (v1.2.4), were directly extracted and transferred into a Seurat object (v5.1.0). This ensured that the UMAP visualization matched the published results exactly. Cell type annotations provided in the original dataset were retained for analysis. These included 25 cell populations such as macrophage subtypes (Mo, AcMo, nrMo), T cells, NK cells, dendritic cells, and epithelial cells. Macrophage subpopulations were defined based on canonical marker gene expression as originally annotated in the manuscript: resident alveolar macrophages (Mo) were identified by expression of *MRC1* and *LYZ*; activated macrophages (AcMo) were distinguished by upregulation of *CCL3* and *CCL4*, and non-resident monocytes (nrMo) were identified by expression of *VCAN*, *FCN1*, and *CCL2* (Marshall et al., 2025). To visualize BCG-induced shifts in cell population composition, overlay UMAP plots were generated for each timepoint (Days 2 and 7). Complement gene expression was further analyzed across cell populations, with a focus on *C3* and *C3AR1*, to assess the role of the complement system in the early mucosal immune response to BCG infection.

### Bulk RNA-sequencing

After training C3-deficient and WT AMs as described, RNA was extracted using an RNeasy Plus kit (QIAGEN, Cat #: 74134). Samples were sent for RNA-sequencing with polyA selection. Samples were prepared according to library kit manufacturer’s protocol, indexed, pooled, and sequenced on an Illumina NovaSeq 6000. Basecalls and demultiplexing were performed with Illumina’s bcl2fastq software and a custom Python demultiplexing program with a maximum of one mismatch in the indexing read. RNA-seq reads were then aligned to the Ensembl release 101 primary assembly with STAR version 2.7.9a (Dobin et al., 2013). Gene counts were derived from the number of uniquely aligned unambiguous reads by Subread:featureCount version 2.0.3 (Liao et al., 2014). Isoform expression of known Ensembl transcripts was quantified with Salmon version 1.5.2 (Patro et al., 2017). Sequencing performance was assessed for the total number of aligned reads, total number of uniquely aligned reads, and features detected. The ribosomal fraction, known junction saturation, and read distribution over known gene models were quantified with RSeQC version 4.0 (Wang et al., 2012).

All gene counts were then imported into the R/Bioconductor package EdgeR (Robinson et al., 2010), and TMM normalization size factors were calculated to adjust for samples for differences in library size. Ribosomal genes and genes not expressed in the smallest group size minus one samples greater than one count-per-million were excluded from further analysis. The TMM size factors and the matrix of counts were then imported into the R/Bioconductor package Limma (Ritchie et al., 2015). Weighted likelihoods based on the observed mean-variance relationship of every gene and sample were then calculated for all samples with the voomWithQualityWeights (Liu et al., 2015) function and were fitted using a Limma generalized linear model with additional unknown latent effects as determined by surrogate variable analysis (Leek and Storey, 2007). The performance of all genes was assessed with plots of the residual standard deviation of every gene to their average log-count with a robustly fitted trend line of the residuals. Differential expression analysis was then performed to analyze for differences between conditions, and the results were filtered for only those genes with Benjamini–Hochberg false-discovery rate adjusted p-values less than or equal to 0.05.

For each contrast extracted with Limma, global perturbations in known Gene Ontology (GO) terms, MSigDb, and KEGG pathways were detected using the R/Bioconductor package GAGE (Luo et al., 2009) to test for changes in expression of the reported log_2_ fold-changes reported by Limma in each term versus the background log_2_ fold-changes of all genes found outside the respective term. The R/Bioconductor package heatmap3 (Zhao et al., 2014) was used to display heatmaps across groups of samples for each GO or MSigDb term with a Benjamini–Hochberg false-discovery rate adjusted p-value less than or equal to 0.05. Perturbed KEGG pathways where the observed log_2_ fold-changes of genes within the term were significantly perturbed in a single-direction versus background or in any direction compared to other genes within a given term with p-values less than or equal to 0.05 were rendered as annotated KEGG graphs with the R/Bioconductor package Pathview (Luo and Brouwer, 2013).

To find the most critical genes, the Limma voomWithQualityWeights transformed log_2_ counts-per-million expression data was then analyzed via weighted gene correlation network analysis with the R/Bioconductor package WGCNA (Langfelder and Horvath, 2008). Briefly, all genes were correlated across each other by Pearson correlations and clustered by expression similarity into unsigned modules using a power threshold empirically determined from the data. An eigengene was then created for each de novo cluster, and its expression profile was then correlated across all coefficients of the model matrix. Because these clusters of genes were created by expression profile rather than known functional similarity, the clustered modules were given the names of random colors, where gray is the only module that has any pre-existing definition of containing genes that do not cluster well with others. These de novo clustered genes were then tested for functional enrichment of known GO terms with hypergeometric tests available in the R/Bioconductor package clusterProfiler (Yu et al., 2012). Significant terms with Benjamini–Hochberg adjusted p-values less than 0.05 were then collapsed by similarity into clusterProfiler category network plots to display the most significant terms for each module of hub genes in order to interpolate the function of each significant module. The information for all clustered genes for each module was then combined with their respective statistical significance results from Limma to determine whether or not those features were also found to be significantly differentially expressed. The data was subsequently processed using Partek Flow, and pathway analysis was done using EnrichR (Chen et al., 2013; Kuleshov et al., 2016; Xie et al., 2021).

### Statistical analyses

Direct comparisons of two isolated groups were analyzed via two-sided unpaired *t*-test, while multiple two-group comparisons were done via *t*-test with the Holm–Šidák correction to control for the family-wise error rate. Analyses of three or more groups against each other were performed using the one-way analysis of variance with Dunnett’s post hoc tests to correct for multiple comparisons. p-values less than 0.05 were considered statistically significant. Statistical analyses were performed with GraphPad Prism 10.0 and independently with R. Data are shown as individual measurements with mean ± SD, while no outliers have been removed.

## Data Availability

The RNASeq data that support the findings are deposited under the accession code GSE281001 (https://www.ncbi.nlm.nih.gov/geo/query/acc.cgi?acc=GSE281001) and are publicly available. Re-used de-identified human data from GSE282132 was used for single-cell RNA sequencing analysis. The source code for this analysis has been uploaded to https://github.com/Kulkarni-Lung-Lab/Re-Analysis-BCG-airway-complement-scRNAseq copy archived at Kulkarni-Lung-Lab, 2026. Raw data used to generate the figures has been made publicly available with this manuscript. The following dataset was generated: EarhartAP LopezAE HernandezJI NallapuA KulkarniHS 2026The C3-C3aR axis modulates trained immunity in alveolar macrophagesNCBI Gene Expression OmnibusGSE28100110.7554/eLife.104977PMC1356151242720115 The following previously published dataset was used: MarshallJ SurakhyM SattiI LiS 2025Early immune responses in the lung and blood following a randomized controlled human inhaled infection with aerosolized attenuated *Mycobacterium bovis* BCG in healthy, BCG-naïve, UK adultsNCBI Gene Expression OmnibusGSE282132

## Additional files

Supplementary file 1. Table of pathways based on Reactome analysis with a list of overlapping genes when comparing downregulated transcripts between trained C3-deficient and wildtype primary alveolar macrophages.

MDAR checklist

Source data 1. The source data includes the raw data used to generate the figures included in the manuscript.
