# Supplementary material for: The C3–C3aR axis modulates trained immunity in alveolar macrophages
Source: eLife. 2026 Sep 10;14:RP104977. doi: 10.7554/eLife.104977 (PMC13561512; doi:10.7554/eLife.104977)
Supplement: Peer review history

# Editor report: eLife Assessment

**DOI:** 10.7554/eLife.104977.3.sa0

**Authors:** Boris Novakovic

**Affiliations:** Murdoch Childrens Research Institute Australia

**Keywords:** Convincing, Important

This study explores how complement protein C3 and its signalling may modulate immune training in alveolar macrophages. The findings are an **important** contribution to the field of trained immunity. The findings are **convincingly** supported by in vivo and ex vivo experiments, encompassing both pharmacological and genetic-based approaches.

# Referee report: Reviewer #1 (Public review)

**DOI:** 10.7554/eLife.104977.3.sa1

[Editors' note: The revised manuscript addressed the concerns of both reviewers, who have concluded that the manuscript is convincing and important. The manuscript can move towards the Version of Record.]

Summary:

This study is built on the emerging knowledge of trained immunity, where innate immune cells exhibit enhanced inflammatory responses upon challenged by a prior insult. Trained immunity is now a very fast-evolving field and has been explored in diverse disease conditions and immune cell types. Earhart and the team approached the topic from a novel angle and was the first to explore a potential link to the complement system.

The study focused on the central complement protein C3 and investigated how its signalling may modulate immune training in alveolar macrophages. The authors first performed in vivo experiments in C57BL mouse models to observe the presence of enhanced inflammation and C3a in BAL fluid following immune training. These changes were then compared with those from C3-deficient mice, which confirmed the involvement of C3a. This trained immunity was further validated in ex vivo experiments using primary alveolar macrophage, which was blunted in C3-deficiency, and, intriguingly, rescued by adding exogenous C3 protein, but not C3a. The genetic-based findings were supported by pharmacological experiments using the C3aR antagonist SB290157. Mechanistically, transcriptomic analyses suggested the involvement of metabolism-linked, particularly glycolytic, genes, which was in agreement with an upregulation of glycolytic flux in WT but not C3-deficient macrophages.

Collectively, these data suggest that C3, possible through engaging with C3aR, contributes to trained immunity in alveolar macrophages.

Strengths:

The conclusions reached were well supported by in vivo and ex vivo experiments, encompassing both genetic-knockout animal models and pharmacological tools.

The transcriptomic and cell metabolism studies provided valuable mechanistic insights.

Weaknesses:

For the in vivo experiments, the histopathological and other inflammatory markers (Fig 1.) were not directly linked to alveolar macrophages by experimental evidence. Other innate immune cells (e.g. dendritic cells, neutrophils) and endothelial cells could also be involved in immune training and contribute to the pathological outcomes. These cells were not examined or mentioned in the study.

For the ex vivo experiments assessing immune training in alveolar macrophages, only the release of selected inflammatory factors were measured. Macrophage activities constitute multiple aspects (e.g. phagocytosis, ROS production, microbe killing), which should also be considered to better depict the effect of trained immunity.

The proposed mechanism of C3 getting cleaved intracellularly then binding to lysosomal C3aR need to be further supported by experimental evidence.

There was an absence of any validation in human-based models.

Comments on the revised version.

The revised manuscript now encompasses a much wider scope and stronger evidence.

The authors have included the re-analysis of a recently published dataset of human volunteers who received aerosolized BCG exposure compared to saline. Although not proven causality, this data helped strengthen the human relevance of the findings presented in this research and directly rationalized the decision to focus on Ams. The persistence of elevated C3/C3aR1 expression to day 7 further supports the idea that complement‑associated reprogramming is not merely an acute inflammatory phenomenon. Whilst it may be outside of the scope of this current study, it would be helpful to clarify in future studies whether other complement components (C5, factor B, factor D) were also modulated in the dataset, to contextualize whether the response is uniquely centered on C3/C3aR1 or part of a broader complement activation program.

The authors have also expanded the functional characterization of trained alveolar macrophages by including phagocytosis and ROS generation measurements. It is intriguing that HKPA training did not markedly alter the phagocytosis and ROS production by alveolar macrophages relative to the control group, however, C3 deficiency significantly dampened these responses in both trained and untrained groups. This reduction is in congruence with the cytokine release data, but there could be other factors involved.

I appreciate the careful revision and much more expansive mechanistic interpretation regarding intracellular C3aR, and that further studies are underway to better understand the cell type-specific, subcellular localization of C3a-C3aR in alveolar macrophages.

Overall, the revised data interpretation and discussion significantly improved in balance and contextualization of the findings.

# Referee report: Reviewer #2 (Public review)

**DOI:** 10.7554/eLife.104977.3.sa2

Earhart et al. investigated the role of the complement system in trained innate immunity (TII) in alveolar macrophages (AM). They used a WT and C3 knockout murine model primed with locally administered heat-killed *P. aeruginosa* (HKPA). Additionally, they employed ex vivo AM training models using C3 knockout mice, where reconstitution of C3 and blockade of C3R were performed. The study concluded that the C3-C3R axis is essential for inducing TII in macrophages in the ex vivo model. The manuscript is well-written and easy to follow.

Comments on revised version.

My concerns have been addressed, and the provided data is convincing supporting the manuscript's claims.

# Author comment: Author response

**DOI:** 10.7554/eLife.104977.3.sa3

**Authors:** Alexander P Earhart, Alberto E Lopez, Josue I Hernandez, Aasritha Nallapu, Deebly Chavez, Sayahi Suthakaran, Brian Yang, Jungheun Hyun, Rafael A Aponte Alburquerque, Marick Starick, Lorena Garnica, Ayse Naz Ozanturk, Rahul Kumar Maurya, Xiaobo Wu, Jeffrey Haspel, Jae Woo Lee, Jaime Hook, Hrishikesh S Kulkarni

**Affiliations:** Washington University in St. Louis St. Louis United States; David Geffen School of Medicine at UCLA Los Angeles United States; David Geffen School of Medicine at UCLA Los Angeles United States; University of California, Los Angeles Los Angeles United States; Icahn School of Medicine at Mount Sinai New York United States; Icahn School of Medicine at Mount Sinai New York United States; David Geffen School of Medicine at UCLA Los Angeles United States; University of California, Los Angeles Los Angeles United States; Washington University in St. Louis St. Louis United States; Washington University in St. Louis St. Louis United States; Washington University in St. Louis St. Louis United States; Washington University in St. Louis St. Louis United States; Washington University in St. Louis St. Louis United States; Washington University School of Medicine St. Louis United States; Washington University in St. Louis St. Louis United States; David Geffen School of Medicine at UCLA Los Angeles United States; Icahn School of Medicine at Mount Sinai New York United States; University of California, Los Angeles Los Angeles United States

The following is the authors’ response to the original reviews.

**Public Reviews:**

**Reviewer #1 (Public review):**

Summary:

This study is built on the emerging knowledge of trained immunity, where innate immune cells exhibit enhanced inflammatory responses upon being challenged by a prior insult. Trained immunity is now a very fast-evolving field and has been explored in diverse disease conditions and immune cell types. Earhart and the team approached the topic from a novel angle and were the first to explore a potential link to the complement system.

The study focused on the central complement protein C3 and investigated how its signalling may modulate immune training in alveolar macrophages. The authors first performed in vivo experiments in C57BL mouse models to observe the presence of enhanced inflammation and C3a in BAL fluid following immune training. These changes were then compared with those from C3-deficient mice, which confirmed the involvement of C3a. This trained immunity was further validated in ex vivo experiments using primary alveolar macrophage, which was blunted in C3-deficiency, and, intriguingly, rescued by adding exogenous C3 protein, but not C3a. The genetic-based findings were supported by pharmacological experiments using the C3aR antagonist SB290157. Mechanistically, transcriptomic analyses suggested the involvement of metabolism-linked, particularly glycolytic, genes, which was in agreement with an upregulation of glycolytic flux in WT but not C3-deficient macrophages.

Collectively, these data suggest that C3, possibly through engaging with C3aR, contributes to trained immunity in alveolar macrophages.

Strengths:

The conclusions reached were well supported by in vivo and ex vivo experiments, encompassing both genetic-knockout animal models and pharmacological tools.

The transcriptomic and cell metabolism studies provided valuable mechanistic insights.

We thank the reviewers for acknowledging the importance of the work.

Weaknesses:

For the in vivo experiments, the histopathological and other inflammatory markers (Figure 1) were not directly linked to alveolar macrophages by experimental evidence. Other innate immune cells (eg. dendritic cells, neutrophils) and endothelial cells could also be involved in immune training and contribute to the pathological outcomes. These cells were not examined or mentioned in the study.

We agree with the suggestions from Reviewer 1 that other cell types such as dendritic cells, neutrophils, and endothelial cells can also be involved in immune training. As the focus of this study was on alveolar macrophages, we specifically focused on these cell types. However,

(1) We have re-analyzed a recently published dataset of human volunteers who received aerosolized BCG exposure compared to saline. We observe that by Day 7, aerosolized BCG exposure alters the expression of *C3* and *C3aR1* in alveolar macrophages in the human bronchoalveolar lavage (BAL) fluid, compared to saline. We have included this new analysis in a revised Figure 1 to clarify why our focus is on investigating the C3-C3aR1 axis in alveolar macrophages.

(2) We have conducted new experiments where we train the mice in vivo, collect the alveolar macrophages, and then provide the second stimulus ex vivo. We observe a similar phenotype in the in vivo trained, ex vivo stimulated alveolar macrophages. We have included this new data in a new Figure S2.

(3) We have updated our Discussion to state “However, we also acknowledge that other immune cells such as dendritic cells and neutrophils, and non-immune cells such as epithelial cells, endothelial cells and fibroblasts can also be involved in immune training (Bigot et al., 2025; Friščić et al., 2021; Moorlag et al., 2020).”

(2) For the ex vivo experiments assessing immune training in alveolar macrophages, only the release of selected inflammatory factors were measured. Macrophage activities constitute multiple aspects (e.g. phagocytosis, ROS production, microbe killing), which should also be considered to better depict the effect of trained immunity.

We agree with the reviewer and have conducted additional experiments to assess immune responses influenced by training in alveolar macrophages. Specifically, we show that in addition to impairing the release of proinflammatory cytokines such as TNFα and IL-6, C3-deficient alveolar macrophages exhibit significantly lower phagocytosis and ROS production compared to WT alveolar macrophages post-training with heat-killed *Pseudomonas aeruginosa*. Results from these additional experiments have been included in new Figure S2.

(3) The proposed mechanism of C3 getting cleaved intracellularly and then binding to lysosomal C3aR needs to be further supported by experimental evidence.

The mechanism of C3 being cleaved intracellularly involves serine protease-dependent cleavage of C3 to C3a and has been experimentally demonstrated previously (Liszewski et al. Immunity 2013; Elvington et al. J Clin Invest 2017). A prior report demonstrated that intracellular C3a interacted with a lysosomal C3aR to promote CD4^+^ T cell survival (Liszewski et al. Immunity 2013). Based on the reviewer’s suggestions, we performed confocal microscopy on alveolar macrophages. Although we clearly observed intracellular colocalization of C3a (using a monoclonal antibody to the neo-epitope) with C3aR, we observed only some colocalization with LAMP1, a lysosomal marker (see Author response image 1). Hence, we will refrain from making comments on how C3 binds to lysosomal C3aR intracellularly in alveolar macrophages, as this may be cell type-specific or stimulation-specific. We have now revised the sentence in the manuscript to remove any references to lysosomal C3aR and now state – “Upon internalization, C3 is cleaved to C3a (Elvington et al., 2017), binds to C3aR, and affects cytokine production in CD4^+^ T cells (Liszewski et al., 2013)”. We have not incorporated the Author response image 1 in the main manuscript as we would like to explore this further to precisely define the subcellular localization of C3a-C3aR in alveolar macrophages, but have provided it for the reviewer to explain the basis of the rewording in the revision.

**Author response image 1.** C3a-C3aR colocalization in mouse *ex vivo* cultured alveolar macrophages (mexAM). mexAMs were harvested and cultured as per the protocol from Gorki et al. (2022). Cells were incubated in a Millicell EZ Slide 8-well glass chamber slide overnight to allow for adherence, then fixed, permeabilized, and incubated with anti-C3a conjugated to AF555 (blue, Hycult HM1072), anti-C3aR conjugated to AF647 (red, Hycult HM1123), and anti-LAMP1 (green, Cell Signaling 99437) overnight at 4°C. Slides were washed 3X in PBS (5 min each) and mounted overnight at 4°C in ProLong Diamond Antifade Mountant with DAPI (white). Images were acquired on a Zeiss LSM 880 confocal microscope at 63X. At least 6 cells per condition imaged. Experiments were conducted in duplicate (technical replicates) and repeated (for biological replicates). Scale bar, 2 μm.

(4) There was an absence of any validation in human-based models.

We acknowledge that the observations need to be validated in human-based models. The focus of our manuscript is on training in alveolar macrophages. Unfortunately, we do not have access to an adequate representation of human alveolar macrophages for our *ex vivo* testing to account for individual-level variation in immune responses. We anticipate this work will form the basis of these future studies. In the interim, we re-analyzed a recently published publicly available dataset of human BAL specimens from human volunteers who underwent aerosolized BCG administration (Marshall et al. Nat Comm 2025). We observe an increase in *C3* and *C3aR1* expression at Day 2, which persists through Day 7 post-training with aerosolized BCG compared to aerosolized saline specifically in human alveolar macrophages. We have included this data in Revised Figure 1. We also validated C3 uptake in alveolar macrophages using precision-cut lung slices from human donors. We have included this additional data in new Supplementary Figure 3.

**Reviewer #2 (Public review):**

Earhart et al. investigated the role of the complement system in trained innate immunity (TII) in alveolar macrophages (AM). They used a WT and C3 knockout murine model primed with locally administered heat-killed *P. aeruginosa* (HKPA). Additionally, they employed ex vivo AM training models using C3 knockout mice, where reconstitution of C3 and blockade of C3R were performed. The study concluded that the C3-C3R axis is essential for inducing TII in macrophages in the ex vivo model. The manuscript is well-written and easy to follow. However, I have the following major concerns.

(1) The secondary challenge to assess the reprogramming of innate cells in the BAL was conducted 14 days after the initial exposure to HKPA. However, no evidence is provided to confirm that homeostasis was re-established following the primary exposure. Demonstrating the resolution of acute inflammation is essential to ensure that the observed responses to the secondary challenge are not confounded by persistent inflammation from the initial exposure.

We thank the reviewer for giving us an opportunity to clarify this point. We have now included additional data from the bronchoalveolar lavage fluid of these mice to show that the levels of protein leaked into the BAL, levels of proinflammatory cytokines (e.g., TNFα, CXCL1) and the neutrophils (all relevant to the acute phase of inflammation) were similar between the untreated and treated wildtype mice. This new data has been included in Figure S1.

(2) In Figure 1D, cytokine production by BAL cells from WT and C3KO mice after HKPA exposure and LPS challenge is shown. However, it is unclear whether the reduced response in trained C3KO mice is due to a defect in trained immunity or an intrinsic inability of C3KO cells to respond to LPS. To clarify this, the response of trained C3KO cells should also be compared to untrained C3KO controls after the LPS challenge. This comparison is necessary to determine if the reduction is specifically related to innate immune memory or a broader impairment in LPS responsiveness. Such control should be included in all ex vivo training and LPS stimulation experiments as well.

We thank the reviewers for their suggestions. We have conducted additional experiments and we observe no significant differences in the BAL cytokine levels between the wildtype and C3-deficient mice post-training in the absence of infection. This new data has been included in Supplementary Figure S1.

Additionally, we came across several manuscripts, including a recent one in eLife as a part of this Series (Gu et al. Elife 2021; Zahalka et al. Mucosal Immunol 2022; Prevel et al Elife 2025) that have done in vivo training followed by an ex vivo challenge. Hence, we have conducted new experiments to compare the response of in vivo HKPA-trained wildtype (WT) and C3-deficient (C3KO) alveolar macrophages compared to untrained AMs after an *ex vivo* LPS challenge. This new data has been included in Figure S2.

(3) The data presented provide evidence of alterations in the functional and metabolic activities of innate cells in the lung, indicating the induction of innate immune memory in a C3-C3R axis-dependent pathway. However, it remains to be established whether such changes can lead to altered disease outcomes. Therefore, the impact of these changes should be demonstrated, for instance, through an infection model to support the claim made in the study that C3 modulates trained immunity in AMs through C3aR signalling.

We acknowledge this is a Limitation of our manuscript. As this is a Short Report, we focused on how C3, via the C3aR, affects the reprogramming of alveolar macrophages. Recent work demonstrated that systemically administered β-glucan induces peripheral trained immunity and aggravates lung injury (Prével et al., 2025), similar to disease in models of periodontitis and arthritis (Haacke et al., 2025). However, training with β-glucan also reduces bleomycin-induced lung fibrosis (Kang et al., 2024). Hence, our ongoing work involves optimizing relevant intrapulmonary exposures to assess how trained immune responses are modulated by the C3a-C3aR axis. We have included the Reviewer’s critique in our revised Discussion as a limitation, while referencing the abovementioned manuscripts.

(4) Figure 3, panels B and C - stats should be shown for comparing WT-HKPA-trained and C3KO HKPA-trained.

These suggestions have been incorporated into Revised Fig 3B and 3C (now Figure 4).

(5) In Figure 4, where the proper untrained C3KO is included, the data presented in Figure 4C show an increase in basal and maximum glycolysis in trained C3KO compared to their untrained control counterparts. Statistical analysis should be provided for this comparison. Based on these data, it appears that metabolic reprogramming occurs even in the absence of C3. Furthermore, C3KO cells intrinsically exhibit reduced glycolytic capacity compared to WT. These observations challenge the conclusions made in the manuscript. Therefore, without the proper control (untrained C3KO) included in all experimental approaches, it is impossible to draw an evidence-based conclusion that the C3-C3R axis plays a role in the induction of innate immune memory.

We have included the statistical comparisons for all the groups in Figure 4C (now Figure 5), as suggested by the reviewer. The data suggests that C3-deficient (C3KO) alveolar macrophages have a blunted metabolic response to training, as compared to C3-sufficient (WT) alveolar macrophages. However, the C3KO cells do not have reduced glycolytic capacity compared to WT in the absence of training. The blunted response in trained C3KO AMs is rescued by exogenous C3, but is then reversed by C3aR antagonism. We have also provided new data/analyses with proper controls (untrained C3KO) in the other Figures (for example, in Figures S1, S2 and 3B&C (now Figure 4)). Taken together, the data would suggest that the effects of C3 in AM reprogramming are C3aR-dependent.

(6) The Results and Discussion sections should be separated, and the results should be thoroughly analyzed in the context of published literature. Separating these sections will allow for a clearer presentation of findings and ensure that the discussion provides a comprehensive interpretation of the data.

We thank the reviewer for this suggestion. The manuscript has been submitted as a Brief Report, and hence, we adhered to the instructions to authors for this format. However, we have added an additional section towards the end of the manuscript based on the Reviewer’s suggestion.

**Recommendations for the authors:**

**Reviewer #1 (Recommendations for the authors):**

It is intriguing that whilst there was a significant elevation of C3a in the cell culture medium of alveolar macrophages, the addition of exogenous C3a failed to rescue the phenotypes of C3-deficient alveolar macrophages. Please help discuss this.

Alveolar macrophages secrete both C3 and proteases, which can cleave C3 to C3a in the supernatant. However, we used the addition of exogenous C3a to compare it to the addition of full-length C3. C3 is internalized by multiple cell types, including alveolar macrophages (as demonstrated in new Figure 3 of our Revised Manuscript) as C3(H_2_O) in comparison to C3a. Hence, we propose that the internalization of C3(H_2_O) provides an intracellular source of C3a (previously reported in Elvington et al. J Clin Invest 2017), which engages with the C3aR to result in alveolar macrophage reprogramming. In comparison, incubating cells with C3a does not exert similar effects. We have included these comments in a separate section towards the end of the manuscript.

Please provide details for the statement "cell-permeable C3aR antagonist (SB290157)" (Figure 3E). Could a paracrine-based mechanism also be at play?

SB290157 does not act selectively on the cell surface, but rather, can also enter cells. Our data, along with previously published reports (Quell et al. J Immunol 2017; Zha et al. Cancer Immunol Res 2019), suggest that C3aR may be intracellular in AMs. However, SB290157 can also block any receptor that may be present on the surface. For this reason, we used exogenous C3a as a way to interrogate surface C3aR signaling, and did not observe significant changes in AM reprogramming with exogenous C3a. However, as this is an indirect approach, we cannot completely rule out a paracrine-based mechanism and have included this limitation in the Discussion section of the revised manuscript.

For Figure 3, please also provide the statistical analysis results for WT versus C3KO HKCA-trained cells. The statistical tests described in the legend for Figure 3D seem to apply to Figure 3E. Please check the labels.

These suggestions have been incorporated into Revised Figure 3 (now Figure 4).
